# Development and internal validation of GENADHECAR: a screening instrument for identifying probable statin non-adherence in patients with ischemic heart disease

**DOI:** 10.3389/fphar.2026.1860683

**Published:** 2026-07-27

**Authors:** Jose A. Quesada, Adriana López-Pineda, Rauf Nouni-García, Alberto Cordero, Álvaro Carbonell-Soliva, Amanda Esquerdo-Arroyo, Avelino Pereira-Exposito, Silvia Guillen-García, Vicente Arrarte, Vicente Bertomeu-González, Andreu Nolasco, Diego Cazorla-Morallón, Julio Osende, Ricardo Nalda-Molina, Domingo Orozco-Beltrán, Concepción Carratalá-Munuera, Vicente Francisco Gil-Guillén

**Affiliations:** 1 Department of Clinical Medicine, Miguel Hernández University (UMH), Sant Joan D'Alacant, Spain; 2 Primary Care Research Center (CIAP), Miguel Hernández University, Sant Joan d'Alacant, Spain; 3 Cardiovascular Research Group (GRINCAVA), Department of Clinical Medicine, Miguel Hernández University of Elche, Alicante, Spain; 4 Institute of Health and Biomedical Research of Alicante (ISABIAL), Alicante, Spain; 5 Research Network on Chronicity, Primary Care, and Health Promotion (RICAPPS), Barcelona, Spain; 6 Health Adherence Research Group (GRINAS), Department of Clinical Medicine, Miguel Hernández University of Elche, Sant Joan D'Alacant, Alicante, Spain; 7 Department of Pathology and Surgery, Miguel Hernández University, Sant Joan D'Alacant, Spain; 8 Cardiology Department, University Hospital Sant Joan D'Alacant, Sant Joan D'Alacant, Spain; 9 Research Unit, General University Hospital of Elda, Alicante, Spain; 10 Cardiology Section, General University Hospital of Elda, Alicante, Spain; 11 Department of Clinical Medicine, Miguel Hernández University of Elche, Sant Joan D'Alacant, Spain; 12 Cardiology Department, General University Hospital Dr. Balmis – ISABIAL, Alicante, Spain; 13 Cardiology Service, Hospital Clínica Benidorm, Alicante, Spain; 14 Department of Clinical Medicine, Miguel Hernández University, Alicante, Spain; 15 Department of Community Nursing, Preventive Medicine, Public Health and History of Science, University of Alicante, San Vicente Del Raspeig, Spain; 16 Cardiology Service, Hospital de Dénia, Alicante, Spain; 17 Clinical Cardiology Unit, HM CIEC, HM Sanchinarro University Hospital, HM Hospitals, Madrid, Spain; HM CIEC, HM Hospitals, Madrid, Spain; 18 HM Hospitals. University Center for Health Sciences, Camilo José Cela University, Madrid, Spain; 19 School of Pharmacy, Miguel Hernández University, San Juan de Alicante, Spain; 20 Alicante Institute for Health and Biomedical Research (ISABIAL-FISABIO Foundation), Alicante, Spain; 21 Research Unit, University Hospital Sant Joan D'Alacant, San Juan de Alicante, Spain

**Keywords:** coronary artery disease, disease management, medication compliance, medication therapy management, secondary prevention, sex factors

## Abstract

**Background:**

No validated and practical tool exists to assess adherence to statins in secondary prevention of cardiovascular disease. Current reference methods for assessing adherence, including plasma drug measurements, are not feasible for routine clinical care or large clinical trials.

**Objective:**

To develop and internally validate a predictive screening instrument for identifying patients at risk of statin non-adherence.

**Methods:**

This multicenter cross-sectional study reports the results of the second phase of the GENADHECAR project in four Spanish hospitals. An initial set of 42 adherence-related items was refined and evaluated in a sample of patients with ischemic heart disease. Responses were compared against a pharmacokinetic reference method (plasma statin concentrations) to assess recent medication intake. A multivariate predictive model of non-adherence was then developed, selecting the best predictors and covariates to be included in the final screening instrument.

**Results:**

A total of 309 patients were included, of whom 49 (15.9%) were classified as non-adherent according to the pharmacokinetic reference method. The final GENADHECAR screening instrument comprised three questionnaire items (use of reminders, forgetfulness, and medication access) and two sociodemographic variables (sex and educational attainment). Internal bootstrap validation yielded an optimism-corrected AUC of 0.694 (95% CI 0.617–0.767), with adequate calibration (Hosmer–Lemeshow p = 0.994). At the selected probability threshold (0.15), sensitivity was 0.73, specificity 0.61, and positive and negative predictive values were 0.26 and 0.92, respectively. Overall, the model showed moderate discrimination and a high capacity to identify patients at low risk of non-adherence.

**Conclusion:**

To our knowledge, GENADHECAR is the first screening instrument with internal validation to identify statin non-adherence using a pharmacokinetic reference method. It offers a brief and practical approach for identifying patients at risk of non-adherence and may help overcome some limitations of conventional self-report methods in this population. Further external validation is needed before routine clinical implementation.

## Introduction

Ischemic heart disease (IHD) consistently ranks as a top global cause of mortality and morbidity; data from the Global Burden of Disease study showed its impact in 2021 was second only to COVID-19, with an estimated 9 million deaths worldwide, along with 188 million lost disability-adjusted life years and 184 million years of life lost to premature mortality (GBD, 2021 Diseases and Injuries Collaborators, 2024; Safiri et al., 2022). These tremendous costs—in lives, health system resources, and lost economic productivity—have fueled decades of scientific advances in primary and secondary prevention of cardiovascular disease (CVD), with statins constituting a cornerstone of pharmacological treatment, complementing behavioral interventions (Adhyaru and Jacobson, 2018; Chou et al., 2022). Yet, up to half of patients prescribed these lipid-lowering drugs stop taking them within a year (Ingersgaard et al., 2020).

Medication adherence is defined as the process, or attitude, by which patients take their medications as prescribed (Vrijens et al., 2012). This complex, multifactorial phenomenon is influenced by several factors related to socioeconomic and cultural determinants (income, place of residence); patients (sex, gender-related factors, beliefs, educational attainment); the disease (symptoms); therapy (polypharmacy, perceived side effects); and the health system (communication, trust) (Gagnon et al., 2017; Yap et al., 2016). High adherence can reduce the risk of a subsequent event by up to 27% in survivors of a previous event (Bansilal et al., 2016)—in whom up to 40% of major cardiovascular events occur (Briffa et al., 2011), while non-adherence reduces efficacy, increases risks, and adds to system inefficiencies (Simpson et al., 2006; Horne et al., 2005). The failure to identify non-adherence may also result in a drug being mistakenly deemed ineffective—including in clinical trials, needlessly intensifying treatments, prompting unnecessary diagnostic tests, or stymying the development of new medicines (Lam and Fresco, 2015).

Understanding and identifying non-adherence is thus a critical component of effective secondary prevention of CVD (Pedretti et al., 2023). Diverse measurement methods are available (López-Pineda et al., 2025). Direct methods (e.g., directly observed therapy, therapeutic drug monitoring) stand out for their diagnostic accuracy, with the determination of plasma drug levels considered one of the most objective approaches to assess recent medication intake. However, indirect techniques (self-report questionnaires, pill counts, pharmacy refill data) are favored for their clinical feasibility and wider adoption in practice. Indirect methods, particularly self-report tools such as the Haynes-Sackett test (Sackett et al., 1978) and Morisky-Green questionnaire (Culig and Leppée, 2014), are widely used but suffer from low sensitivity, poor predictive value, and lack of validation against objective pharmacokinetic reference methods or for specific diseases like IHD and treatments, including statins (López-Pineda et al., 2025). Moreover, existing instruments do not consider sex- and gender-related determinants, despite robust evidence showing that non-adherence behaviors and barriers differ between women and men (Goldstein et al., 2016). To address these gaps, the present study aimed to develop and internally validate a screening instrument for identifying probable statin non-adherence in patients with IHD, using plasma statin concentrations as a pharmacokinetic reference method. The development process incorporated a sex- and gender-informed perspective based on findings from previous qualitative and quantitative phases of the GENADHECAR project.

## Materials and methods

### Study design

This multicenter, cross-sectional study was conducted during the second phase of the 5-year GENADHECAR project, whose aim was to design and internally validate a screening instrument for identifying medication non-adherence in secondary prevention of IHD. The study protocol has been published elsewhere (Lopez-Pineda et al., 2024). The present study took place from July 2022 to April 2025 and builds on the qualitative and quantitative results from phase 1, published elsewhere (Carbonell-Soliva et al., 2024; Carbonell-Soliva et al., 2025), which identified the dimensions associated with lower adherence. Participant recruitment and data collection for the validation phase were conducted between November 2022 and July 2024. All participants signed written, informed consent upon recruitment.

Based on the results of the psychometric analysis, phase 2 procedures were adapted from those initially described in the study protocol (Lopez-Pineda et al., 2024) to orient the instrument toward a predictive screening instrument rather than a structured questionnaire (Carbonell-Soliva et al., 2025). Moreover, logistical limitations precluded validating the instrument for identifying non-adherence to acetylsalicylic acid treatment, as the systematic collection of biological samples for this drug proved unfeasible. The study was reported in accordance with the Transparent Reporting of a Multivariable Prediction Model for Individual Prognosis or Diagnosis (TRIPOD) statement.

### Study population and setting

The validation phase included adult patients (≥18 years) attending the cardiology units in four Spanish hospitals for routine check-up. This cohort constituted the study population for the criterion validation and model development analyses described below. Inclusion criteria were: diagnosis of IHD, pharmacological treatment for secondary prevention of cardiovascular disease for at least the last 12 months (acetylsalicylic acid, atorvastatin, or rosuvastatin), and the ability to communicate in Spanish. Patients were excluded if they had a treatment interruption in the previous year, a neurological or psychological condition that prevented completion of the study data collection form, a life expectancy of less than 1 year, a disease that compromised their capacity for self-assessment, physical or clinical conditions that could affect treatment adherence, or significant difficulties reading or writing.

The planned sample size was 440 patients (Lopez-Pineda et al., 2024). A pilot study was performed in 40 patients (20 women and 20 men) from one study center to test recruitment, completion of the data collection form, biological sampling, and analytical procedures.

### Instrument development and evaluation

**FIGURE 1:**
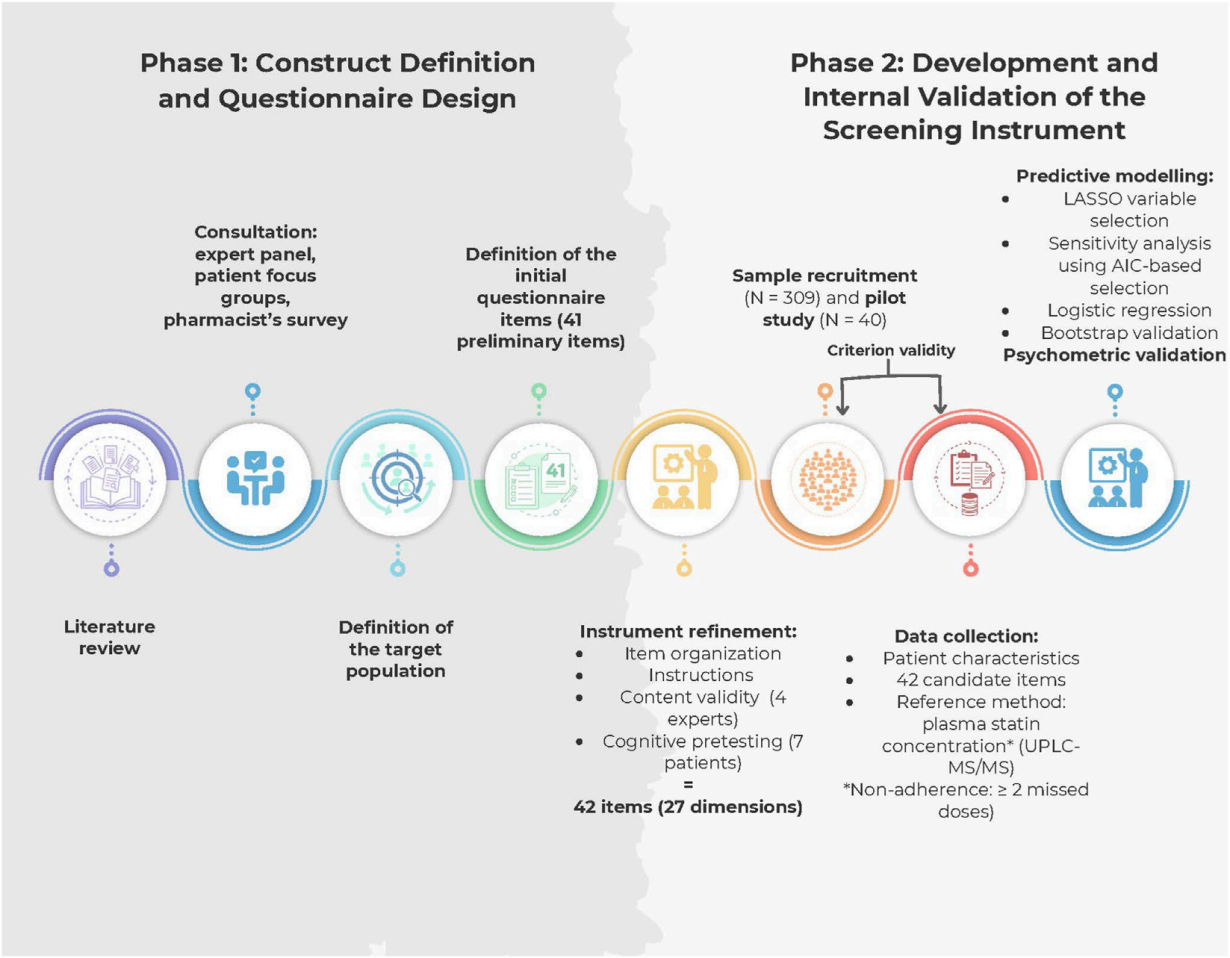
Development and evaluation workflow of the GENADHECAR predictive screening instrument. Two-phase development process. Phase 1 included construct definition, identification of adherence-related dimensions, and generation of candidate items. Phase 2 included content validity assessment, cognitive pretesting, psychometric evaluation, criterion validation against a pharmacokinetic reference method based on plasma statin concentrations, and predictive modeling using logistic regression with bootstrap validation. The final predictive screening instrument was derived from a broad set of candidate predictors, including adherence-related items and patient characteristics.

The GENADHECAR instrument was developed and evaluated through an iterative process integrating conceptual refinement and empirical evaluation. The items and dimensions identified in phase 1^20^ were the basis for the procedures conducted in phase 2, described below and in Figure 1.

#### Organization of the items

Forty-one initial items were arranged on a 5-point Likert scale and grouped by thematic domains to optimize clarity and flow (Argimón Pallas and Jiménez Villa, 2019; Casas et al., 2003; Morales Vallejo, 2011). Items assessing opinions and experiences were separated to reduce fatigue. A control item was added to assess response reliability. Response options were formatted vertically and consistently (Casas et al., 2003; Morales Vallejo, 2011). Final formatting decisions followed recommendations from a panel of psychologists (Morales Vallejo, 2011). Detailed procedures are provided in Supplementary Material 1.

#### Instructions for item administration

The instructions used plain language and emphasized the importance of honest, complete responses (Casas et al., 2003; Rosellini and Brown, 2021). Two psychologists reviewed the text to ensure clarity, consistency, and readability.

#### Content validity

A panel of four experts (two in questionnaire methodology, two in cardiovascular disease) independently assessed the relevance, clarity, and understandability of each item, as well as the appropriateness of the response scale (Sireci and Geisinger, 1992), using a structured online form (July 2022). Items with ≥2 “disagree” ratings were revised. The full content-validity protocol is described in Supplementary Material 1.

#### Assessment of understandability: cognitive pretesting

Cognitive interviews were conducted with 7–10 adults with IHD using think-aloud and verbal probing techniques to detect comprehension issues (Morales Vallejo, 2011; León and Montero, 2020). Participants represented different age groups and educational levels. Interviews were conducted at Miguel Hernández University (September–November 2022). Findings were used to refine wording, order, and clarity. Detailed procedures are in Supplementary Material 1.

#### Criterion validity

To assess the criterion validity of the study instrument, an objective pharmacokinetic procedure based on the determination of plasma concentrations of statins (atorvastatin or rosuvastatin) was used as a pharmacokinetic reference method for recent drug intake, acknowledging the inherent variability of drug concentration measurements.

Blood samples were collected during routine visits on the same day as completion of the study data collection form. Participants were asked about their usual statin administration schedule. Most reported taking their statin in the evening, while blood samples were generally collected the following morning as part of routine clinical care. However, the interval between statin intake and blood sampling was not standardized. Samples were processed and stored following a standard protocol in all participating centers: EDTA-K2 tubes, refrigeration at 2 °C–8 °C, centrifugation at 3,000 rpm for 10 min at 4 °C within 30 min of collection, plasma separation (1 mL), −80 °C storage, and shipment on dry ice to an external laboratory. Ultra-high-performance liquid chromatography–tandem mass spectrometry was used for quantification.

Due to the instability of atorvastatin, its active metabolite (atorvastatin lactone) was quantified using a validated algorithm (Kristiansen et al., 2019). For rosuvastatin, expected steady-state concentrations were derived from theoretical pharmacokinetic curves. Following the approach proposed by Kristiansen et al. and consistent with previous adherence research, non-adherence was operationally defined as omission of at least two consecutive statin doses, with the aim of identifying clinically meaningful treatment interruptions rather than isolated missed doses. Adherence classification was not based on individualized post-dose concentration predictions according to the exact dosing-to-sampling interval. Instead, non-adherence was defined as plasma concentrations below predefined statin-specific thresholds consistent with omission of the two most recent doses. For rosuvastatin, the thresholds were <1.07 ng/mL (5 mg/day), <1.44 ng/mL (10 mg/day), and <2.46 ng/mL (20 mg/day). For atorvastatin, the validated algorithm based on atorvastatin and atorvastatin lactone concentrations classified patients as non-adherent when the dose-adjusted concentration was <0.05 nmol/L. This approach assumes a simplified pharmacokinetic profile and does not explicitly account for intra- and interindividual variability in drug absorption, metabolism, and disposition, which may have introduced some degree of exposure misclassification. The analytical methodology is described in detail in Supplementary Material 2. The resulting binary classification (non-adherent vs. adherent/partially adherent) served as the reference classification for all model performance analyses.

#### Psychometric evaluation: construct validity and reliability

An exploratory factor analysis was performed to identify latent dimensions and assess item grouping using conceptual and statistical criteria. Internal reliability was evaluated for each dimension and for the final set of selected items, to determine whether the resulting instrument exhibited psychometric properties consistent with a latent-construct measure.

### Study variables

We collected demographic and socioeconomic variables (age, sex, education, employment, living situation), self-perceived health, behavioral risks (smoking, alcohol, physical activity), anthropometric and clinical data (body mass index [BMI], vital signs, comorbidities), and pharmacological treatments (prescriptions, drug intake patterns). The primary outcome variable was statin non-adherence status, operationalized as a binary variable (non-adherent vs. adherent/partially adherent) based on plasma drug concentrations.

### Statistical analysis

Missing data were imputed using median values for quantitative variables and the most frequent category for qualitative variables. Given the low proportion of missing data, a simple imputation approach was considered appropriate. Imputation was performed on the full sample and was not conditioned on adherence status. Responses to the study items were dichotomized on an item-by-item basis as suggestive of adherence or non-adherence, based on expert consensus to manage the odd number of response options and establish clinically relevant risk thresholds. For the descriptive analysis, frequencies were calculated for qualitative variables, and means and standard deviation for quantitative variables. Fisher’s exact test was used to assess associations between non-adherence status (pharmacokinetic reference method) and qualitative variables, and the Chi-squared test was used to assess associations between non-adherence status and the 42 study items.

Given the high number of potential predictors relative to the sample size, a variable selection process was performed using penalized logistic regression (LASSO) to construct a predictive model of non-adherence, starting with the 42 candidate items and all prespecified covariates. The optimal penalty parameter, lambda, was estimated through 10-fold cross-validation. For predictors with non-zero coefficients in the LASSO regression, a logistic model was fitted to estimate standard errors, confidence intervals, and p-values. As a sensitivity analysis in the variable selection process, predictors with a p-value of association with non-adherence status <0.2 were also preselected. With these candidates, a stepwise variable selection process was performed based on the Akaike information criterion (Akaike, 1974). Interactions between sex and the predictors retained in the final model were subsequently evaluated. The areas under the ROC curve of the models estimated by both selection processes were compared. The final model was selected considering the results of both approaches, model discrimination, and the clinical relevance of the retained predictors.

Goodness-of-fit was evaluated using the likelihood ratio test (LRT), calibration by the Hosmer-Lemeshow test, and discrimination by calculating the area under the receiver operating curve (AUC) and its 95% CI, both in the original sample and in 1000 bootstrap simulations, to estimate optimism-corrected performance. The optimism-corrected calibration intercept and slope were estimated using 1000 bootstrap simulations for the final model, as well as the Brier score and its 95% confidence interval. The calibration plot was also calculated. For the final model, sensitivity, specificity, predictive values and likelihood ratios were also estimated for different cutoff points. The optimal cut-off point was defined as the threshold maximizing the Youden Index (sensitivity + specificity −1). A decision curve analysis was performed to evaluate clinical net benefit according to the different probability cut-off points.

Finally, the construct structure was subjected to an exploratory factor analysis on the final items of each model, using tetrachoric correlation matrices due to the dichotomous nature of the items, checking the multivariate normal distribution by Mardia’s test, and the adequacy of the factor analysis by Bartlett’s test and the Kaiser-Meyer-Olkin (KMO) test. The internal consistency of the items of each model was estimated using Cronbach’s alpha (Cronbach, 1951) and McDonald’s omega (McDonald, 1999) coefficients, with their respective 95% CIs. All analyses were performed using R (version 4.5.1).

## Results

### Preliminary testing and item refinement

After ordering the 41 items identified in the previous phase of the study (Carbonell-Soliva et al., 2024), content validity was confirmed for 38 items (92.7%). Two items were considered relevant by three experts, and one item by a single expert. Clarity was confirmed for 26 items (63.4%) by all four experts; 12 items by three experts, and three items by two experts. Response scales were adequate for 28 items (68.3%) according to all experts; 13 items (31.7%) were agreed upon by three experts. Minor lexical-syntactic adjustments were applied to 15 items (36.6%) to improve clarity and reduce redundancy, examples of these modifications and their rationale are provided in Supplementary Table S1.

Cognitive interviews were conducted with seven patients (four men and three women), aged 49–68 years to assess comprehension, format, and instructions. As shown in Supplementary Table S2, participants represented different age groups and educational levels, supporting the evaluation of item comprehension across diverse patient profiles. Feedback led to minor wording adjustments in five items and the addition of a new dimension (“fear”) within the “patient” domain, expressed through the item *My disease frightens me*. This concept had initially been excluded, as it was previously considered part of the dimension “apprehension,” which expert reviewers had associated primarily with concerns about treatment and potential side effects. However, qualitative feedback from patients supported distinguishing between fear of the disease itself and apprehension toward medication effects. The final set of candidate items for model development comprised 42 items across five domains and 27 dimensions (Supplementary Material 2).

### Characteristics of the sample

A total of 309 patients were analyzed. Missing data were infrequent, with a maximum of 3.5% missing values for any explanatory variable and 1.2% for any questionnaire item; these values were imputed as described in the Methods section. The final sample had a mean age of 66.1 years (range 30–92); 32.7% (n = 101) were women, 5.2% (n = 16) were foreigners, 45.6% (n = 141) had primary education or less, 60.2% (n = 186) were retired, 49.9% (n = 154) had a net household income of less than 1500€/month, 16.2% (n = 50) lived alone, 62.1% (n = 192) had good or very good self-perceived health, 11.3% (n = 35) were current smokers and 57.3% (n = 177) ex-smokers, and 14.9% (n = 46) had a sedentary lifestyle (Table 1). The main comorbidities were dyslipidemia (72%, n = 225), hypertension (66.3%, n = 205), and diabetes (31%, n = 96); the full distribution of comorbid conditions is presented in Supplementary Table S3. Apart from the statins that all participants were taking (atorvastatin: 56.0%, n = 173, rosuvastatin: 44.0%, n = 136), the most frequent treatments were angiotensin-converting enzyme (ACE) inhibitors (77.7%, n = 240) and beta-blockers (66%, n = 204); a complete description of concomitant pharmacological treatments is provided in Supplementary Table S4. On average, participants were taking 7.3 daily pills (median 7.0), spread out over a mean 2.5 administration times (median 3.0) (Table 1).

**TABLE 1:** Sample characteristics (n = 309).

Variables	Missings n (%)*	​	n	%
Sex	0 (0%)	Female	101	32.7%
	​	Male	208	67.3%
Nationality	0 (0%)	Spanish	293	94.8%
	​	Other	16	5.2%
Educational attainment	0 (0%)	< Primary school	14	4.5%
	​	Primary school	127	41.1%
	​	Secondary school	83	26.9%
	​	Higher education	85	27.5%
Employment status	0 (0%)	Employed	83	26.9%
	​	Unemployed	27	8.7%
	​	Retired	186	60.2%
	​	Homemaker	13	4.2%
Net monthly household income	0 (0%)	<800 €	50	16.2%
	​	800€–1499€	104	33.7%
	​	1500€–3,000€	96	31.1%
	​	>3,000 €	55	17.8%
	​	NA	4	1.3%
Living situation	0 (0%)	Lives alone	50	16.2%
	​	Does not live alone	259	83.8%
Caregiver	1 (0.3%)	No	282	91.3%
	​	Yes	27	8.7%
Self-perceived health	0 (0%)	Very poor	4	1.3%
	​	Poor	17	5.5%
	​	Fair	96	31.1%
	​	Good	153	49.5%
	​	Very good	39	12.6%
Tobacco use	0 (0%)	Current smoker	35	11.3%
	​	Ex-smoker	177	57.3%
	​	Never smoker	97	31.4%
Alcohol intake	0 (0%)	Abstainer	126	40.8%
	​	Once weekly	120	38.8%
	​	Several times weekly	42	13.6%
	​	Daily	21	6.8%
Leisure-time activity	0 (0%)	Sedentary	46	14.9%
	​	Occasional physical activity	115	37.2%
	​	Several times a month	112	36.2%
	​	Several times a week	36	11.7%
Hypertension	0 (0%)	No	104	33.7%
​	​	Yes	205	66.3%
Diabetes	0 (0%)	No	213	68.9%
​	​	Yes	96	31.1%
Dyslipidemia	0 (0%)	No	84	27.2%
​	​	Yes	225	72.8%
​	​	​	Mean	SD
Age, years	2 (0.6%)	​	66.1	10.0
Body mass index, kg/m^2^	11 (3.5%)	​	28.2	4.2
Systolic blood pressure, mmHg	4 (1.2%)	​	131.2	18.9
Diastolic blood pressure, mmHg	4 (1.2%)	​	75.9	10.6
Resting heart rate, beats per min	5 (1.6%)	​	68.0	11.8
N pills per day	1 (0.3%)	​	7.3	3.5
N administration times/day	3 (0.9%)	​	2.5	0.8

* Number of missings before imputation.

According to the pharmacokinetic reference method, an estimated 49 patients (15.9%, 95% CI 11.7%–19.7%) were classified as non-adherent to statins, with similar proportions in men and women (16.3% vs. 14.9%, p = 0.74). Low educational attainment was the only variable significantly associated with non-adherence (p = 0.003); results for all sociodemographic and clinical variables are shown in Supplementary Table S5.

### Item performance and model development

All 42 items were dichotomized as indicative of adherence or non-adherence; the response-grouping criteria applied to each item are detailed in Supplementary Table S6. Supplementary Table S7 presents the distribution of the resulting dichotomized response categories for all items. The lowest variability was observed for items Q3 (1.6%), Q2 (1.9%) and Q33 (4.2%). Table 2 shows the items with a p-value of less than 0.2 in the chi-squared test of association with non-adherence, with items Q20 and Q33 reaching statistical significance at a 95% confidence level. A complete list of item-adherence associations is provided in Supplementary Table S8.

**TABLE 2:** Candidate adherence-related items with a relation (p < 0.2) to non-adherence to statins treatment, as measured against a pharmacokinetic reference method.

Candidate adherence-related item	Adherence according to references method		p-value*
	Partially adherent/adherent	Non-adherent	
	(n, %)	(n, %)	
*(Q5) My disease frightens me*			
Strongly agree, agree, neither agree nor disagree	153 (81.8)	34 (18.2)	0.17
Disagree, strongly disagree	107 (87.7)	15 (12.3)	​
*(Q6) Skipping my medication every once in a while does not have serious consequences for my health*			
Strongly agree, agree, neither agree nor disagree	116 (80.0)	29 (20.0)	0.061
Disagree, strongly disagree	144 (87.8)	20 (12.2)	​
*(Q8) If I do not take my medication correctly, I may suffer from complications*			
Strongly agree, agree	231 (85.2)	40 (14.8)	0.16
Neither agree nor disagree, disagree, strongly disagree	29 (76.3)	9 (23.7)	​
*(Q12) I know what the medications I take are for*			
Strongly agree, agree	234 (90.0)	47 (95.9)	0.19
Neither agree nor disagree, disagree, strongly disagree	26 (10.0)	2 (4.1)	​
*(Q17) Taking medication just once a day would make it easier for me to take it correctly*			
Strongly agree, agree, neither agree nor disagree	173 (66.5)	27 (55.1)	0.12
Disagree, strongly disagree	87 (33.5)	22 (44.9)	​
*(Q20) Reminder systems (for example, pill boxes, notes, alarms) are useful for remembering to take my medication*			
Strongly agree, agree	183 (87.1)	27 (12.9)	0.035
Neither agree nor disagree, disagree, strongly disagree	77 (77.8)	22 (22.2)	​
*(Q26) I know what blood pressure levels I should have to keep my heart disease under control*			
Strongly agree, agree	230 (88.5)	40 (81.6)	0.19
Neither agree nor disagree, disagree, strongly disagree	30 (11.5)	9 (18.4)	​
*(Q33) I forget to take my medicine*			
Always, almost always, sometimes	8 (61.5)	5 (38.5)	0.023
Seldom, never	252 (85.1)	44 (14.9)	​
*(Q36) Picking up my medication from the pharmacy is difficult for me*			
Always, almost always, sometimes	20 (74.1)	7 (25.9)	0.13
Seldom, never	240 (85.1)	42 (14.9)	​
*(Q40) In the last month, I have felt bad or have had an unexpected symptom due to the medications I take*			
Always, almost always, sometimes	26 (10.0)	8 (16.3)	0.19
Seldom, never	234 (90.0)	41 (83.7)	

* Chi-squared test.

Table 3 shows the final logistic regression model to estimate the probability of statin non-adherence. The significant predictors were items Q20, Q33, and Q36, and educational attainment was a significant covariate. A significant interaction was detected between item Q36 and sex, such that it was only significant in women. These predictors represented distinct adherence-related dimensions: Q20 (use of reminders), Q33 (forgetfulness), and Q36 (medication access).

**TABLE 3:** Multivariable logistic regression for non-adherence to statins treatment.

​	Beta	Standard error	OR	95% CI	p-value
Intercerpt	−2.004	0.372	​	​	​
(Q20) reminder systems (for example, pill boxes, notes, alarms) are useful for remembering to take my medication. (*Neither agree nor disagree, disagree or Strongly disagree*)	0.838	0.343	2.312	(1.180–4.528)	0.014
(Q33) I forget to take my medicine. (*always, almost always, sometimes*)	1.746	0.643	5.732	(1.625–20.21)	0.007
Educational attainment					
*≤ Primary*	0	​	1	​	​
*Secondary*	−1.820	0.551	0.162	(0.055–0.477)	0.001
*Higher education*	−0.709	0.398	0.492	(0.226–1.074)	0.075
Interaction: Sex × (Q36) picking up my medication from the pharmacy is difficult for me					
*Women × seldom, never*	0	​	1	​	​
*Women × always, almost always, sometimes*	2.896	0.870	18.093	(3.289–99.53)	0.001
*Men × seldom, never*	0	​	1	​	​
*Men × always, almost always, sometimes*	0.004	0.692	1.003	(0.258–3.895)	0.995
Sample size	​	​	​	n = 309	
Non-adherent	​	​	​	n = 49	
LRT chi-square (p-value)	​	​	​	32.33 (<0.001)	
AUC (95% CI)	​	​	​	0.731 (0.653–0.808)	
AUC (95% CI)*	​	​	​	0.694 (0.617–0.767)	
Hosmer-lemeshow test (p-value)	​	​	​	0.994	
Calibration intercept: Apparent/corrected*	​	​	​	0.000/−0.367	
Calibration slope: Apparent/corrected*	​	​	​	1.000/0.750	
Brier score (95% CI)	​	​	​	0.117 (0.08–0.138)	

AUC, area under the receiver operating curve; CI, confidence interval LRT, likelihood ratio test; OR, odds ratio.

* Bootstrap 1000 simulations.

The model achieved an AUC of 0.731 with a 95% CI (0.653–0.808). Internal bootstrap validation (1000 resamples) yielded an optimism-corrected AUC of 0.694 with a 95% CI (0.617–0.767). Calibration was adequate according to the Hosmer–Lemeshow test (p = 0.994). Bootstrap validation yielded an optimism-corrected calibration intercept of −0.367 and a calibration slope of 0.750. The Brier score was 0.117 with a 95% CI (0.08–0.138). The calibration plot (Supplementary Figure S1) indicated acceptable agreement between predicted and observed probabilities.

### Performance of the final screening instrument

The predictive indicators were calculated at different cutoffs expressing the probability of non-adherence (Table 4). The optimal threshold according to the Youden index was 0.15, yielding a sensitivity of 0.73 (95% CI 0.61–0.85), a specificity of 0.61 (95% CI 0.55–0.67), and positive and negative predictive values of 0.26 and 0.92, respectively, with slightly better performance in women (Supplementary Tables SS9, S10 show results stratified by sex). The final GENADHECAR screening instrument comprises five components: (1) sex, (2) educational attainment, (3) ‘Reminder systems are useful for remembering to take my medication’, (4) ‘I forgot to take my medications’, (5) ‘Picking up my medication from the pharmacy is difficult for me’. The calculation procedure and application of the final screening instrument are described below. The decision curve analysis (Supplementary Figure S2) showed that the model provided a higher net benefit than the “treat all” and “treat none” strategies across a wide range of threshold probabilities. At the selected threshold probability of 0.15, the model maintained a positive net benefit, supporting its potential use as a screening instrument.

**TABLE 4:** Predictive indicators for the final model.

Cutoff	Overall detection accuracy	Youden index	Sensitivity (95% CI)		Specificity (95% CI)		PPV (95% CI)		NPV (95% CI)		LR+ (95% CI)		LR− (95% CI)	
0.10	0.52	0,30	0.84	(0.74–0.94)	0.46	(0.40–0.52)	0.23	(0.17–0.29)	0.94	(0.90–0.98)	1.56	(1.32–1.84)	0.35	(0.18–0.67)
**0.15**	**0.63**	**0,34**	**0.73**	**(0.61–0.85)**	**0.61**	**(0.55–0.67)**	**0.26**	**(0.19–0.33)**	**0.92**	**(0.88–0.96)**	**1.87**	**(1.49–2.35)**	**0.44**	**(0.27–0.71)**
0.20	0.78	0,29	0.45	(0.31–0.59)	0.84	(0.80–0.88)	0.34	(0.22–0.46)	0.89	(0.85–0.93)	2.81	(1.85–4.26)	0.65	(0.50–0.84)
0.25	0.80	0,27	0.39	(0.25–0.53)	0.88	(0.84–0.92)	0.38	(0.25–0.51)	0.88	(0.84–0.92)	3.25	(2.01–5.26)	0.69	(0.55–0.87)
0.30	0.81	0,28	0.39	(0.25–0.53)	0.89	(0.85–0.93)	0.40	(0.26–0.54)	0.89	(0.85–0.93)	3.55	(2.16–5.83)	0.69	(0.55–0.87)
0.35	0.85	0,14	0.16	(0.06–0.26)	0.98	(0.96–1.00)	0.57	(0.31–0.83)	0.86	(0.82–0.90)	8.00	(2.9–22.08)	0.86	(0.76–0.97)
0.40	0.85	0,12	0.14	(0.04–0.24)	0.98	(0.96–1.00)	0.58	(0.30–0.86)	0.86	(0.82–0.90)	7.00	(2.31–21.17)	0.88	(0.78–0.99)
0.45	0.85	0,11	0.12	(0.03–0.21)	0.99	(0.98–1.00)	0.67	(0.36–0.98)	0.86	(0.82–0.90)	12.00	(3.10–46.44)	0.89	(0.80–0.99)
0.50	0.85	0,11	0.12	(0.03–0.21)	0.99	(0.98–1.00)	0.75	(0.45–1.05)	0.86	(0.82–0.90)	12.00	(2.50–57.68)	0.89	(0.80–0.99)
0.55	0.85	0,09	0.10	(0.02–0.18)	0.99	(0.98–1.00)	0.71	(0.37–1.05)	0.85	(0.81–0.89)	10.00	(2.00–50.04)	0.91	(0.83–1.00)

CI, confidence interval; LR+, positive likelihood ratio; LR−, negative likelihood ratios; NPV, negative predictive value; PPV, positive predictive value. Bold values indicate the selected cutoff (0.15) and its corresponding diagnostic performance measures used in the final model.

### Application of the GENADHECAR screening instrument

The final GENADHECAR screening instrument incorporates three self-report items (Q20, Q33, and Q36), educational attainment, and sex. The coding of predictors included in the model is presented in Table 5.

**TABLE 5:** Coding of predictors included in the GENADHECAR screening instrument.

Predictor		Coding
Q20	Reminder systems (for example, pill boxes, notes, alarms) are useful for remembering to take my medication	1 = neither agree nor disagree, disagree, or strongly disagree; 0 = agree or strongly agree
Q33	I Forget to take my medicine	1 = always, almost always, or sometimes; 0 = seldom or never
Q36	Picking up my medication from the pharmacy is difficult for me	1 = always, almost always, or sometimes; 0 = seldom or never
ES	Secondary educational attainment	1 = yes; 0 = No
EH	University educational attainment	1 = yes; 0 = No
SEX	Sex	1 = man; 0 = woman

The probability of non-adherence is estimated using the following logistic regression equation:

$$\text{logit}\left(p\right)=‐2+0.838\;Q20+1.746\;Q33+2.896\;Q36+0.463\;\text{SEX}‐1.82\;E_{S}‐0.709\;E_{H}‐2.892\;Q36\;\text{SEX}$$

where *p* is the probability of non-adherence. The predicted probability is calculated as:

$$P\left(nonadherent\;patient\right)=\frac{1}{1+e^{-logit\left(p\right)}}$$

Patients are classified as being at increased risk of non-adherence when the estimated probability exceeds 0.15. This threshold corresponds to the maximum value of the Youden index and yielded a sensitivity of 0.73 and a specificity of 0.61.

As an example, consider a woman (SEX = 0) with university education (ES = 0; EH = 1) who agrees that reminder systems are useful for remembering to take medication (Q20 = 0), reports never forgetting to take medication (Q33 = 0), and reports difficulties obtaining medication from the pharmacy (Q36 = 1). For this patient:

$$logit\left(p\right)=-2+0.838*0+1.746*0+2.896*1+0.463*0-1.82*0-0.709*1-2.892*1*0=0.187$$

$$P\left(nonadherent\;patient\right)=\frac{1}{1+e^{-0.187}}=0.547$$

Therefore, this patient would be classified as being at increased risk of non-adherence because the estimated probability (0.547) exceeds the predefined threshold of 0.15.

### Construct validity and reliability

Exploratory factor analysis did not reveal a stable factorial structure. Multivariate normality was not met (Mardia test p < 0.001), and the adequacy of the correlation matrix for factor analysis was poor (Bartlett test p < 0.001; KMO<0.50). Internal reliability was low. These findings indicate limited internal consistency and support the interpretation of GENADHECAR as a predictive screening instrument for non-adherence risk rather than as a psychometric scale designed to measure latent constructs.

## Discussion

This study developed a screening instrument capable of identifying patients with IHD at risk of non-adherence to statin therapy. From a broad set of candidate predictors, including adherence-related items and patient characteristics, a model was constructed comprising three questionnaire items related to the use of reminders, forgetfulness, and access to medication, together with two sociodemographic variables (sex and educational attainment). This model showed moderate discriminatory performance, adequate calibration, and reasonable performance as a screening instrument for identifying patients at increased risk of non-adherence to statins.

This new instrument is particularly relevant in the current context, as the tools available to measure adherence to statins—and to chronic treatments in general—have significant methodological limitations. Recent systematic reviews have found that most studies use indirect methods that are rarely validated against pharmacokinetic reference methods and do not consider potential sex- and gender-related determinants or specific clinical contexts (López-Pineda et al., 2025; Nouni-García et al., 2025; Schnorrerova et al., 2024). Moreover, large-scale meta-analytic evidence shows that adherence to statins remains suboptimal, with pooled estimates around 60%–65% and substantial variability across populations, influenced by demographic and clinical factors (Basios et al., 2025). Researchers often use self-report questionnaires designed for other indications, without specific validation for statins (López-Pineda et al., 2025; Mantila et al., 2022). To our knowledge, only one adaptation of the Measure of Adherence to Treatment (MAT) instrument has been proposed for this pharmacological group, with significant limitations (Veloso et al., 2023). Against this backdrop, the tool we propose offers an innovative approach: a brief, 5-item instrument developed within a sex- and gender-informed framework, based on barriers reported by patients and healthcare professionals, and internally validated against a pharmacokinetic reference method. These comparative advantages are also relevant when contrasting with randomized clinical trials, where the methods used to measure adherence are highly heterogeneous and rarely validated (Mantila et al., 2022).

Although exploratory factor analysis did not identify a clear dimensional structure—which limits the interpretation of GENADHECAR as a psychometric instrument designed to measure latent constructs—the selected items retained individual predictive value for identifying patients at risk of non-adherence. Therefore, the instrument should be viewed as a screening instrument for identifying patients at increased risk of non-adherence to statins rather than as a psychometric instrument designed to measure latent constructs or explain underlying behavioral mechanisms. Its brevity—only five components—enhances its feasibility for rapid screening in overloaded clinical settings, where brief and easy-to-use assessments are required. In our study, the model achieved a sensitivity of 0.73 and a specificity of 0.61 for detecting non-adherence, consistent with the performance observed at the selected cutoff. In comparison, Kristiansen et al. (Kristiansen et al., 2021) evaluated three commonly used tools against a pharmacological reference method and found considerably lower sensitivities: 40% for weekly self-reporting, 32% for the Morisky Medication Adherence Scale (MMAS-8) scale (Morisky et al., 2008), and 22% for Gehi’s question (Gehi et al., 2005). Although these tools showed high specificity (94%–96%), all of them fail to identify a substantial proportion of truly non-adherent patients, limiting their usefulness for early detection, although comparisons should be interpreted cautiously due to differences in study design and populations. Furthermore, none were developed or validated specifically for statins use, and the original publication of the MMAS-8 scale has been retracted, raising additional questions about its validity. The performance characteristics of GENADHECAR suggest that its greatest practical value lies in identifying patients at low risk of non-adherence. The high NPV (0.92) indicates that patients classified as low risk are unlikely to be classified as non-adherent according to the pharmacokinetic reference method. Conversely, the relatively low PPV (0.26) means that many patients identified as being at risk of non-adherence will ultimately prove adherent. Nevertheless, the instrument identifies a subgroup with a higher probability of non-adherence than the baseline prevalence observed in the study population, supporting its use as a screening tool to prioritize further assessment or low-cost adherence-promoting interventions. Therefore, the instrument should not be used to confirm in isolation non-adherence or guide treatment decisions. The MAT questionnaire, partially adapted for this population, showed modest internal consistency (Cronbach’s alpha of 0.66) and lacks studies demonstrating criterion validity against objective methods (Veloso et al., 2023).

The three dimensions included in the final model—forgetfulness, use of reminders, and access to medication—align with factors supported by robust, consistent evidence. Forgetfulness is repeatedly the most frequent cause of non-adherence reported by patients and professionals (Carbonell-Soliva et al., 2025; Kvarnström et al., 2021; Shin et al., 2025). The absence of support strategies to organize medication intake, such as pill boxes or alarms, has been identified as an important and modifiable factor (Kvarnström et al., 2021). Although there is less evidence regarding access challenges, these frequently appear as obstacles in qualitative studies and in contexts involving co-payment or logistical problems. Previous studies (Grimmond et al., 2023; Compton et al., 2010) have documented how barriers like physical distance, pharmacy opening hours, and lack of transport can significantly reduce prescription collection rates and, by extension, adherence. These findings support the clinical relevance of the selected dimensions despite their limited internal consistency as a unified scale. Likewise, the inclusion of sex and educational attainment as variables in the final model is supported by consistent evidence associating female sex (Arshed et al., 2024; Lopes and Santos, 2021) and low educational attainment (Lopes and Santos, 2021) with non-adherence, although their effects may vary depending on the predominant barriers in each group.

In contrast, despite the moderate but relatively consistent evidence showing that younger age is associated with higher non-adherence (Lopes and Santos, 2021), age was not selected in the final model. This result could be explained by the age distribution of the sample or by the lower predictive capacity of this variable compared to other included dimensions. Other dimensions with higher empirical support, such as concerns about adverse effects, polypharmacy, the relationship with the healthcare professional, or mood, were likewise not selected by the final model (Shin et al., 2025; Chung et al., 2018). Their exclusion does not obviate their clinical relevance but is rather suggestive of a lower discriminatory power in this sample or their lack of independent contribution to the final model. The difference between clinical versus predictive value reflects the multifactorial complexity of adherence. This highlights the distinction between variables associated with non-adherence in explanatory models and those with sufficient discriminatory capacity for prediction. In this sense, the instrument developed provides a useful screening tool, focused on factors directly associated with the risk of non-adherence, but it does not replace more integrative approaches when seeking to understand the underlying causes of patient behavior.

Although our data did not show significant differences in the prevalence of non-adherence between men and women, sex and a sex-specific interaction were retained in the final model. Female sex has often been associated with a higher risk of non-adherence (Arshed et al., 2024; Lopes and Santos, 2021), whereas other studies have not identified a significant independent association in multivariable analyses (Berglund et al., 2013). These inconsistencies suggest that sex may influence adherence indirectly through differences in perceived barriers and contextual factors rather than through a direct effect on adherence itself. In the previous qualitative phase of the GENADHECAR project (Carbonell-Soliva et al., 2024), for example, men tended to point to dosage frequency and low perceived severity as key factors, while women more frequently highlighted mood-related factors and difficulties accessing healthcare resources. Consistent with these findings, the final model retained a significant interaction between sex and difficulties accessing medication, indicating that this barrier was more strongly associated with non-adherence among women. Although the present study did not directly measure gender-related social determinants, this finding is consistent with the possibility that social and contextual factors influence adherence behaviours differently in women and men. Therefore, the gender-informed nature of GENADHECAR should be understood primarily in terms of its conceptual framework and development process, which explicitly explored potential sex- and gender-related barriers to adherence, rather than as evidence that the final model comprehensively captures gender constructs. Future studies should further investigate the mechanisms underlying the observed sex-specific association and evaluate whether the incorporation of explicit gender-related determinants improves model performance.

This study, and the resulting clinical tool, has several strengths that merit highlighting. It is based on a robust design, including a co-creation process involving clinicians, pharmacists, and patients; the incorporation of sex- and gender-related considerations during instrument development; and efforts to recruit a sample representative of both male and female patients. In addition, it adopts a novel and specific approach to identifying patients at risk of statin non-adherence in patients with IHD, validated against a pharmacokinetic reference method. Importantly, the use of pharmacokinetic measurements as a reference represents a major strength compared with indirect adherence measures, allowing for a more objective classification of recent medication intake. These features may enhance the potential clinical applicability of the instrument and address several limitations of previous approaches, although external validation remains necessary.

However, some limitations should be considered when interpreting the results. First, the final sample size was smaller than initially planned (309 rather than 440 patients), which may have reduced statistical power and increased the uncertainty of the estimated effects. This limitation is particularly relevant for subgroup and interaction analyses, as interaction terms generally require larger sample sizes than main-effect analyses. Accordingly, the sex-specific association observed for difficulty obtaining medication from the pharmacy should be interpreted with caution, as the corresponding interaction estimate may be less stable than the main model coefficients. In addition, internal bootstrap validation showed some degree of optimism, reflected by a reduction in discrimination after correction and an optimism-corrected calibration slope below 1, suggesting moderate overfitting. Despite our efforts, complete sex parity was not achieved. The tool was validated only in patients with IHD treated with statins (atorvastatin and rosuvastatin) in hospitals in Spain, which may limit the generalizability of the findings to other populations, settings, or lipid-lowering therapies. Its application in other countries or cultural contexts would require appropriate cross-cultural and linguistic adaptation and validation. In addition, the characteristics of the Spanish healthcare system, including partial reimbursement of pharmacological treatments, may influence adherence behaviors and limit comparability with other healthcare systems. External validation in independent cohorts is therefore required before routine implementation of the model can be recommended.

Although the use of a pharmacokinetic reference method to assess non-adherence represents a major strength compared with indirect approaches, it also has limitations. Statin dose intensity was not incorporated into the predictive model and may warrant consideration in future studies. Blood samples were obtained at a single time point, reflecting recent rather than longitudinal adherence. Moreover, plasma statin concentrations may be influenced by the timing of the last dose relative to blood sampling, statin dose, organ function, concomitant medications, and other sources of pharmacokinetic variability. Non-adherence was defined as the omission of the two most recent statin doses, based on previously published criteria (Chung et al., 2018) and intended to identify clinically meaningful treatment interruptions likely to disrupt steady-state drug exposure, rather than isolated missed doses. For atorvastatin, the cutoff was derived from empirical data, whereas for rosuvastatin it was based on a theoretical pharmacokinetic model. This model-based approach does not account for intra- and interindividual variability in drug exposure, nor for the known multicompartmental behavior of rosuvastatin. Consequently, the rosuvastatin cutoff should be interpreted as an approximation of non-adherence rather than a definitive threshold and may be subject to some degree of misclassification. However, within our study population, the prevalence of non-adherence was of a similar magnitude in patients treated with atorvastatin and rosuvastatin, which provides some reassurance regarding the internal validity of the classification. Finally, due to logistical constraints, the instrument could not be validated for acetylsalicylic acid, limiting its applicability across the full range of secondary prevention therapies. Although the lack of a stable factorial structure and the low internal reliability limit its use as a psychometric instrument, this does not preclude its intended use as a brief predictive screening tool for identifying patients at increased risk of statin non-adherence.

These findings have important clinical implications. The 5-item GENADHECAR screening instrument can be completed in approximately 2 min, facilitating the rapid identification of patients at risk of non-adherence to statin therapy. If confirmed in external validation studies, this could support timely adherence-focused interventions, additional assessment, simplified regimens, or tailored support strategies. The tool may also have potential applications in clinical trials evaluating lipid-lowering therapies. It may be useful in adherence research, particularly given the limited availability of instruments specifically developed and validated for assessing statin non-adherence in patients with IHD. Nevertheless, as GENADHECAR estimates the probability of non-adherence rather than directly measuring medication-taking behavior, it should preferably be used in combination with other adherence assessment methods when accurate classification is required.

Future research should assess its external validity in other populations and healthcare settings, including primary care, and support its cross-cultural and linguistic adaptation for use in different countries. Its applicability to other pharmacological treatments and patient groups should also be explored, as well as its potential for identifying individual barriers to adherence that could be addressed through targeted interventions.

In conclusion, the GENADHECAR screening instrument represents a brief approach for identifying patients at risk of statin non-adherence. The instrument demonstrated encouraging performance in this internally validated cohort and may have potential utility in clinical practice and research settings, although external validation is required before routine implementation.

## Data Availability

The datasets generated and analyzed in this study are publicly available in Zenodo under the title “GENADHECAR PROJECT” (Quesada JA, 2025) and can be accessed via the following DOI: https://doi.org/10.5281/zenodo.17790828.

## Group members of Nantes DIVAT Consortium

Collaborators (GENADHECAR Group) GENADHECAR Group: José J Mira-Solves (Miguel Hernandez University), M Virtudes Pérez Jover (Miguel Hernandez University), José Verdú Soriano (Alicante University), Leticia Fernández Friera (HM Hospitales), Beatriz Martin García (HM Hospitales), Eduardo Paya Mora (Elda University Hospital), Joaquina Díaz-Torres (Elda University Hospital), Francisco González LLopis (Elda University Hospital), Raquel Ajo (Alicante Hospital) Pablo Martínez Pérez P (Miguel Hernandez University), Kamila Cheikh-Moussa (Alicante University), Jose María Castelano (HM Hospitales).
