# Supplementary material for: Development and internal validation of GENADHECAR: a screening instrument for identifying probable statin non-adherence in patients with ischemic heart disease
Source: Front Pharmacol. 2026 Jul 27;17:1860683. doi: 10.3389/fphar.2026.1860683 (PMC13454762; doi:10.3389/fphar.2026.1860683)
Supplement: Supplementary file 1 [file Supplementaryfile1.docx]

Supplementary Material

Table of contents

Supplemental methods	2

Supplementary Material 1. Detailed description of the organization of the items, content validity and cognitive pretesting procedures.	2

Supplementary Material 2. Direct method for measuring adherence to statin treatment.	3

Supplemental results	6

Supplementary Material 3. Questionnaire instructions and items, classified by domain and dimension, and the response scale (English and Original Spanish-language versions).	6

Supplemental tables	15

Supplementary Table 1. Content validity: initial items and their dimensions, proposed modifications and final items.	15

Supplementary Table 2. Characteristics of patients undergoing cognitive interviews	17

Supplementary Table 3. Prevalence of comorbidities among participants (n=309)	18

Supplementary Table 4. Ongoing pharmacological treatments in participants (n = 309)	19

Supplementary Table 5. Prevalence of non-adherence to statins, as estimated using the gold standard, according to sociodemographic and clinical variables	20

Supplementary Table 6. Expert consensus dichotomization of item responses: assignment of Likert scale (5-point) responses to the category "suggestive of non-adherence" *.	22

Supplementary Table 6, *continued*. Dichotomization of item responses according to frequency*.	23

Supplementary Table 7. Frequency of dichotomized responses for each questionnaire item.	24

Supplementary Table 8. Association between dichotomized questionnaire items and adherence to statin treatment measured by the gold standard method.	26

Supplementary Table 9. Measures of diagnostic accuracy for preditive model in men	28

Supplementary Table 10. Measures of diagnostic accuracy for preditive model in women	28

Supplemental figures	29

Supplementary Figure 1. Calibration plot for predictive model	29

Supplementary Figure 2. Decision curve for prediction model	30

References	31

# Supplemental methods

## Supplementary Material 1. Detailed description of the organization of the items, content validity and cognitive pretesting procedures.

- Organization of the items

The questionnaire was structured to ensure logical coherence, readability, and respondent engagement. The 41 preliminary items were arranged in a clear sequence, with responses captured on a five-point Likert scale. The most accessible items were placed at the beginning to capture participants’ interest and encourage truthful responses^1–3^, while more difficult questions were placed in the middle and end to avoid the initial stress that could condition subsequent responses. Next, items were grouped thematically: first by domain and dimension to optimize the flow and internal consistency, favor respondents’ concentration, and simplify the interpretation of the results^1,2^; and second into items eliciting opinions versus experiences to reduce cognitive fatigue and improve visual presentation. Following the recommendation of the panel of psychologists specialized in questionnaire design, a control item was added at the end of the opinion questions to verify the reliability of the responses^3,4^. The items were numbered consecutively, and the response options were presented in vertical format to facilitate data entry^1^. The presentation of the response options was consistent, and no questions were divided between pages^5^.

- Content validity

Content validity was assessed by an expert panel following a systematic approach^6^; the panel was composed of two experts in questionnaire research and two specialists in CVD. Each expert independently assessed the relevance, clarity, and understandability of each item, as well as the appropriateness of the response scale, using a Google Forms survey (July 2022) with 45 questions. Survey responses were collected using a three-point Likert scale (agree, neutral, disagree). Adjustments were made when at least two experts selected “disagree” on any item.

- Assessment of understandability: cognitive pretesting

Cognitive interviews were conducted with patients to identify possible spelling errors, ambiguities, and other aspects that could make it difficult to understand and complete the questionnaire^7,8^. For this evaluation, we planned to recruit 7 to 10 adults with IHD who had been in treatment for at least one year, selected from previous phases of the GENADHECAR study ^9^ and through clinical networks. Ideally, this group included people of different ages and educational levels, with a similar proportion of men and women. The interviews took place at Miguel Hernández University from September to November 2022. Participants read each item silently and expressed their interpretations using the think-aloud method, then the interviewer used the verbal probing technique to check their understanding and identify doubts^7,8^. Participants were also asked about the length and order of the items. All observations were recorded, and the pertinent adjustments were made to improve clarity. The changes derived from these interviews were incorporated into the final version of the tool.

## Supplementary Material 2. Direct method for measuring adherence to statin treatment.

**Overview**

Patients meeting inclusion criteria were recruited during routine cardiology follow-up visits at the participating hospitals. Blood samples were collected at the same time as the completion of the adherence questionnaire.

**Analytical procedure**

The direct method consisted of quantifying the plasma concentration of atorvastatin or rosuvastatin, depending on the prescribed drug. Samples were centrifuged at low temperature, stored at –80 °C, and subsequently transported on dry ice to a certified reference laboratory.
Quantification was performed by ultra-high performance liquid chromatography coupled with tandem mass spectrometry (UHPLC–MS/MS) using a validated analytical algorithm. Given the instability of atorvastatin, its concentration was determined together with that of its active metabolite, atorvastatin lactone, following the method described by Kristiansen et al^10.^

**Definition of low adherence**

Low adherence was defined as the absence of at least two consecutive daily doses of the prescribed statin. Specific cutoffs were derived separately for rosuvastatin and atorvastatin based on theoretical pharmacokinetic modeling and validated empirical algorithms, respectively.

**Rosuvastatin**

Cutoff values were estimated using theoretical pharmacokinetic curves under steady-state conditions according to the following equation:

C_t_=C_max_⋅e^−ktC^

where *t* is time (hours) and *k* is the elimination constant calculated as:

k=ln2 / T_1/2_

Parameters were derived from published pharmacokinetic data according to the dose administered (*Table S1*).

**Table S1**. Theoretical curve parameters according to rosuvastatin dose^11^.

| Dose | C_max_ (ng/mL) | T ½ (hours) ^12^ | Cutoff (ng/mL) |
| --- | --- | --- | --- |
| 5 mg | 4 .6 | 19 | < 1 .07 |
| 10 mg | 6 .2 | 19 | < 1 .44 |
| 20 mg | 10 .6 | 19 | < 2 .46 |

Figure S1 illustrates attainment of steady-state concentrations after three consecutive doses of 20 mg, while Figure S2 shows the progressive decline in plasma levels after the last dose, reaching the cutoff concentration (2.46 ng/mL) at approximately 48 hours.

**Figure S1**. Stabilization of the concentration of rosuvastatin at a dose of 20 mg.

C (ng/mL)

| Time (hours) | C |
| --- | --- |
| 3 | 9.50112 |
| 6 | 8.51616 |
| 9 | 7.63331 |
| 12 | 6.84198 |
| 15 | 6.13268 |
| 18 | 5.49692 |
| 21 | 4.92707 |
| 24 | 4.41629 |
| 24 | 15.0163 |
| 27 | 13.4596 |
| 30 | 12.0643 |
| 33 | 10.8136 |
| 36 | 9.69256 |
| 39 | 8.68775 |
| 42 | 7.78711 |
| 45 | 6.97984 |
| 48 | 6.25625 |
| 51 | 16.8563 |
| 54 | 15.1088 |
| 57 | 13.5425 |
| 60 | 12.1386 |
| 63 | 10.8802 |
| 66 | 9.75227 |
| 69 | 8.74127 |
| 72 | 7.83508 |
| 75 | 7.02284 |
| 78 | 17.6228 |
| 81 | 15.7959 |
| 84 | 14.1584 |
| 87 | 12.6906 |
| 90 | 11.375 |
| 93 | 10.1958 |
| 96 | 9.13881 |
| 99 | 8.19141 |
| 102 | 7.34222 |
| 105 | 17.9422 |
| 108 | 16.0822 |
| 111 | 14.415 |
| 114 | 12.9206 |
| 117 | 11.5812 |
| 120 | 10.3806 |
| 123 | 9.30443 |
| 126 | 8.33986 |
| 129 | 7.47529 |

C dead

| Time (hours) | C (ng/mL) |
| --- | --- |
| 3 | 14.1584 |
| 6 | 12.6906 |
| 9 | 11.375 |
| 12 | 10.1958 |
| 15 | 9.13881 |
| 18 | 8.19141 |
| 21 | 7.34222 |
| 24 | 6.58107 |
| 27 | 5.89882 |
| 30 | 5.2873 |
| 33 | 4.73918 |
| 36 | 4.24788 |
| 39 | 3.80751 |
| 42 | 3.41279 |
| 45 | 3.059 |
| 48 | 2.74188 |
| 51 | 2.45763 |
| 54 | 2.20285 |
| 57 | 1.97449 |
| 60 | 1.7698 |
| 63 | 1.58633 |
| 66 | 1.42188 |
| 69 | 1.27447 |
| 72 | 1.14235 |
| 75 | 1.02393 |
| 78 | 0.917778 |
| 81 | 0.822633 |
| 84 | 0.737353 |
| 87 | 0.660913 |
| 90 | 0.592397 |
| 93 | 0.530985 |
| 96 | 0.475939 |
| 99 | 0.426599 |
| 102 | 0.382375 |
| 105 | 0.342735 |
| 108 | 0.307204 |
| 111 | 0.275357 |
| 114 | 0.246811 |
| 117 | 0.221225 |
| 120 | 0.198291 |
| 123 | 0.177734 |
| 126 | 0.159309 |
| 129 | 0.142794 |
| 132 | 0.127991 |
| 135 | 0.114722 |
| 138 | 0.102829 |

| Time (hours) | C (ng/mL) |
| --- | --- |
| 48 | 0 |
| 48 | 16 |

**Figure S2**. Decline in rosuvastatin concentration with a 20 mg dose.

**Atorvastatin**

Pharmacokinetic parameters for atorvastatin differ by dose and are less stable; therefore, concentration thresholds were derived from the validated empirical model described by Kristiansen et al. (2019)^10^. This model combines atorvastatin and its lactone metabolite concentrations as follows:

1. Unit conversion:

Atorvastatin:	(nmol/L) = (ng/mL) × 1000 / 558.64

Atorvastatin Lactone:	(nmol/L) = (ng/mL) × 1000 / 540.62

2. Sum of both analytes:

Total Atorvastatin = atorvastatin + lactone

3. Dose adjustment:

Adjusted concentration=Total atorvastatin / Dose (mg)

Patients were classified as non-adherent if the adjusted concentration was < 0.05 nmol/L, corresponding to omission of two or more consecutive doses.

According to Kristiansen et al., this cutoff yields a sensitivity of 100% and specificity of 92% when samples are handled at low temperatures, as in this study.

**Sensitivity analysis**

A sensitivity analysis was conducted to confirm the consistency between the theoretical cutoffs estimated for rosuvastatin and the empirical cutoffs for atorvastatin.

# Supplemental results

## Supplementary Material 3. Questionnaire instructions and items, classified by domain and dimension, and the response scale (English and Original Spanish-language versions).

**English version of the** **questionnaire, with instructions and items, classified by domain and dimension, and the response scale.**

**Instructions:** Patients are to complete the questionnaire themselves, without the help of a healthcare professional, while waiting for their appointment. The questionnaire is completely anonymous, so patients are encouraged the following questions honestly. Select only one answer. Select the answer that best reflects your first impression. Do not spend too much time on each question; your first impression is probably the one that fits best for your. The questionnaire will take approximately 10-15 minutes to complete.

|  | | | **Scale of responses according to level of agreement** | | | | | |
| --- | --- | --- | --- | --- | --- | --- | --- | --- |
| **Domain** | **Dimension** | **Item** | **Strongly agree** | **Agree** | **Neither agree nor disagree** | **Disagree** | | **Strongly disagree** |
| Patient | Beliefs | 1. My heart disease is serious. |  |  |  |  | |  |
|  |  | 2. Taking care of myself is important for managing my heart disease. |  |  |  |  | |  |
|  | Educational attainment | 3. I understand why I take my medications. |  |  |  |  | |  |
|  | Depression/cognitive impairment | 4. I feel sad or depressed. |  |  |  |  | |  |
|  | Fear | 5. My disease frightens me. |  |  |  |  | |  |
|  | Perception of risk | 6. Skipping my medication every once in a while doesn’t have serious consequences for my health. |  |  |  |  | |  |
|  |  | 7. When I’m feeling better, I stop taking my medication or I take less of it. |  |  |  |  | |  |
|  |  | 8. If I don’t take my medication correctly, I may suffer from complications. |  |  |  |  | |  |
|  | Sociofamiliar circumstances | 9. Nobody helps me take my medication. |  |  |  |  | |  |
|  | Apprehension | 10. If I stop taking my medications, it’s because I believe they may be harmful to me. |  |  |  |  | |  |
|  | Beliefs | 11. Medications are a business for pharmaceutical companies. |  |  |  |  | |  |
|  | Informacion/misinformation | 12. I know what the medications I take are for. |  |  |  |  | |  |
| Treatment | Number of pills | 13. Taking several pills a day makes it difficult to take my medication properly. |  |  |  |  | |  |
|  | Access | 14. Buying the medications I need for my heart condition is a big financial strain on me. |  |  |  |  | |  |
|  |  | 15. Sometimes I run out of medication. |  |  |  |  | |  |
|  | Format | 16. The change in the format (color, shape, and/or brand name) of my medication confuses me when it comes to taking my medication. |  |  |  |  | |  |
|  | Dosage | 17. Taking medication just once a day would make it easier for me to take it correctly. |  |  |  |  | |  |
|  | Perception of benefit | 18. The medications I take work well for me and are really helping me feel better and stay healthy. |  |  |  |  | |  |
|  |  | 19. Taking my medication prevents my heart disease from getting worse. |  |  |  |  |  |  |
|  | Reminder systems | 20. Reminder systems (for example pill boxes, notes, alarms) are useful for remembering to take my medication. |  |  |  |  | |  |
|  | Naming medication | 21. I know the names of all the medications I take. |  |  |  |  | |  |
|  | Adverse effects | 22. Taking my medication causes some unpleasant side effects. |  |  |  |  | |  |
| Relationship between health professional and patient | Shared decisión-making | 23. Health professionals keep my preferences and opinions in mind to treat my disease. |  |  |  |  | |  |
|  | Trust | 24. I have a good relationship with the cardiology service as a whole. |  |  |  |  | |  |
| Disease | Management | 25. I know what cholesterol levels I should have to keep my heart disease under control. |  |  |  |  | |  |
|  |  | 26. I know what blood pressure levels I should have to keep my heart disease under control. |  |  |  |  | |  |
| Health system | Consultation time | 27. My disease is given enough time in the healthcare system. |  |  |  |  | |  |
|  | Multidisciplinary team | 28. Another health professional (nurse, pharmacist, family doctor) helps me to follow my treatment correctly. |  |  |  |  | |  |
|  | Use of services | 29. Since I’ve had heart problems, I visit my cardiologist at least once a year. |  |  |  |  | |  |
| Patient | Beliefs | 30. My heart condition is not dangerous to my health. |  |  |  |  | |  |
|  | | | **Scale of responses according to frequency** | | | | | |
|  |  |  | **Always** | **Almost always** | **Sometimes** | **Seldom** | **Never** | |
| Disease | Lack of symptoms | 31. At present, my heart disease is causing, for example, tachycardia, chest pain, shortness of breath, dizziness, general discomfort, or fainting. |  |  |  |  |  | |
| Patient | Forgetfulness | 32. I leave medication somewhere other than my home (e.g., work, car, purse, wallet) in case I forget to take it at home. |  |  |  |  |  | |
|  |  | 33. I forget to take my medicine. |  |  |  |  |  | |
|  |  | 34. When I leave my usual environment (for example, if I am away from home, on vacations, out of town), I forget to take my medication. |  |  |  |  |  | |
|  | Beliefs | 35. I prefer natural remedies to conventional medicines. |  |  |  |  |  | |
| Treatment | Access | 36. Picking up my medication from the pharmacy is difficult for me. |  |  |  |  |  | |
|  |  | 37. Visiting my cardiologist is difficult for me, for example, because it’s difficult to get an appointment, they don’t answer the phone, it is far from my home, etc. |  |  |  |  |  |  |
|  | Dosage | 38. I take medication more than once a day |  |  |  |  |  | |
|  | Size | 39. I have trouble swallowing my pills. |  |  |  |  |  | |
|  | Adverse effects | 40. In the last month, I have felt bad or have had an unexpected symptom due to the medications I take. |  |  |  |  |  | |
|  | Sexual health | 41. I notice that the medication I take negatively affects my sex life. |  |  |  |  |  | |
| Relationship between health professional and patient | Health education | 42. My cardiologist explains to me how I should take my medication. |  |  |  |  |  | |

**Original Spanish-language questionnaire instructions and items, classified by domain and dimension, and the response scale.**

**Instrucciones:** El cuestionario será autocumplimentado por el paciente sin ayuda del profesional sanitario mientras espera a pasar la consulta. El cuestionario es totalmente anónimo, responda con sinceridad las siguientes cuestiones. Marque una sola respuesta. Señale la respuesta que mejor refleje su primera impresión. No emplee demasiado tiempo con cada pregunta, probablemente su primera impresión es la que mejor le identifique. El tiempo aproximado para cumplimentar el cuestionario será de 10-15 minutos.

|  | | | **Escala de respuesta de grado de acuerdo** | | | | | |
| --- | --- | --- | --- | --- | --- | --- | --- | --- |
| **Dominio** | **Dimensión** | **ítem** | **Totalmente de acuerdo** | **De acuerdo** | **Ni acuerdo ni desacuerdo** | **En desacuerdo** | | **Totalmente en desacuerdo** |
| Paciente | Creencias | 1. La enfermedad del corazón que tengo es grave. |  |  |  |  | |  |
|  |  | 2. Cuidarse es importante para controlar mi enfermedad del corazón. |  |  |  |  | |  |
|  | Nivel educativo | 3. Entiendo por qué tomo mis medicinas. |  |  |  |  | |  |
|  | Depresión/deterioro cognitivo | 4. Me siento triste o deprimido. |  |  |  |  | |  |
|  | Miedo | 5. Mi enfermedad me genera miedo. |  |  |  |  | |  |
|  | Percepción de riesgo | 6. Dejar de tomar la medicación alguna vez no tiene efectos graves sobre mi salud. |  |  |  |  | |  |
|  |  | 7. Cuando me encuentro mejor dejo de tomar mi medicación o tomo menos cantidad. |  |  |  |  | |  |
|  |  | 8. Si no tomo la medicación de forma adecuada, puedo sufrir complicaciones. |  |  |  |  | |  |
|  | Situación sociofamiliar | 9. No hay nadie que me ayude a tomar mi medicación. |  |  |  |  | |  |
|  | Temor | 10. Si dejo de tomar la medicación es porque creo que puede perjudicarme. |  |  |  |  | |  |
|  | Creencias | 11. Los medicamentos son un negocio de las farmacéuticas. |  |  |  |  | |  |
|  | Información/desinformación | 12. Conozco bien para qué son las medicinas que tomo. |  |  |  |  | |  |
| Tratamiento | Número de pastillas | 13. Tomar varias pastillas al día hace que sea complicado tomar la medicación adecuadamente. |  |  |  |  | |  |
|  | Accesibilidad | 14. Comprar los medicamentos, que necesito para mi enfermedad del corazón, me supone un gran esfuerzo económico |  |  |  |  | |  |
|  |  | 15. A veces me quedo sin medicación |  |  |  |  | |  |
|  | Formato | 16. El cambio en el formato (de color, forma y/o nombre comercial) de mi medicación me confunde a la hora de tomar mi medicación. |  |  |  |  | |  |
|  | Posología | 17. Tomar la medicación una sola vez al día facilitaría que me la tomara adecuadamente. |  |  |  |  | |  |
|  | Percepción del beneficio | 18. Los medicamentos que tomo me van bien y realmente me están ayudando a sentirme mejor y a mantenerme sano. |  |  |  |  | |  |
|  |  | 19. Tomar mi medicación evita que empeore mi enfermedad del corazón |  |  |  |  |  |  |
|  | Sistemas de recuerdo | 20. Es útil el uso de sistemas de recuerdo (por ejemplo: pastilleros, notas, alarmas…) para recordar tomar mi medicación. |  |  |  |  | |  |
|  | Enumerar la medicación | 21. Conozco los nombres de los medicamentos que tomo. |  |  |  |  | |  |
|  | Efectos adversos | 22. Tomar mi medicación me produce algún efecto secundario molesto. |  |  |  |  | |  |
| Relación prof. Sanitario/paciente | Toma de decisiones compartida | 23. Los profesionales sanitarios tienen en cuenta mis preferencias y opiniones para tratar mi enfermedad. |  |  |  |  | |  |
|  | Confianza | 24. Tengo buena relación con el servicio de cardiología en su conjunto. |  |  |  |  | |  |
| Enfermedad | Grado de control | 25. Conozco qué cifras de colesterol son las adecuadas para tener mi enfermedad del corazón controlada. |  |  |  |  | |  |
|  |  | 26. Conozco qué cifras de tensión arterial son las adecuadas para tener mi enfermedad del corazón controlada. |  |  |  |  | |  |
| Sistema de salud | Tiempo de consulta | 27. El sistema sanitario dedica suficiente tiempo en consultas a mi patología. |  |  |  |  | |  |
|  | Equipo multidisciplinar | 28. Otro profesional sanitario (enfermero, farmacéutico, médico de cabecera) me ayuda para que siga mi tratamiento correctamente. |  |  |  |  | |  |
|  | Uso de servicios | 29. Desde que tengo problemas de corazón visito a mi cardiólogo por lo menos una vez al año. |  |  |  |  | |  |
| Pacientes | Creencias | 30. Mi enfermedad de corazón no es peligrosa para mi salud. |  |  |  |  | |  |
|  | | | **Escala de respuesta de nivel de frecuencia** | | | | | |
|  |  |  | **Siempre** | **Casi siempre** | **Algunas veces** | **Rara vez** | **Nunca** | |
| Enfermedad | Falta de síntomas | 31. En la actualidad, mi enfermedad del corazón presenta (por ejemplo: taquicardia, dolor en el pecho, falta de aire, mareos, malestar general, desmayo…). |  |  |  |  |  | |
| Paciente | Olvido | 32. Dejo medicación en otro lugar diferente a mi domicilio (por ejemplo: trabajo, coche, bolso, cartera, etc.) por si olvido tomarla en casa. |  |  |  |  |  | |
|  |  | 33. Se me olvida tomar la medicación. |  |  |  |  |  | |
|  |  | 34. Cuando salgo de mi entorno habitual (por ejemplo: estar fuera de casa, vacaciones, viajes…) se me olvida tomar la medicación. |  |  |  |  |  | |
|  | Creencias | 35. Prefiero tomar los remedios naturales a las medicinas convencionales. |  |  |  |  |  | |
| Tratamiento | Accesibilidad | 36. Recoger mi medicación de la farmacia me resulta difícil. |  |  |  |  |  | |
|  |  | 37. Visitar a mi cardiólogo me resulta difícil (por ejemplo, por: dificultad conseguir cita, no cogen el teléfono, está lejos de mi domicilio…). |  |  |  |  |  |  |
|  | Posología | 38. Tomo medicamentos más de una vez al día. |  |  |  |  |  | |
|  | Tamaño | 39. Tengo dificultades para tragar mis pastillas. |  |  |  |  |  | |
|  | Efectos adversos | 40. En el último mes, debido a los medicamentos que tomo, he sentido malestar o he tenido algún síntoma inesperado. |  |  |  |  |  | |
|  | Salud sexual | 41. Noto que la medicación que tomo afecta negativamente a mi vida sexual. |  |  |  |  |  | |
| Relación prof. Sanitario/paciente | Educación sanitaria | 42. Mi cardiólogo me explica la forma adecuada en la que debo tomar mi medicación. |  |  |  |  |  | |

# Supplemental tables

## Supplementary Table 1. Content validity: initial items and their dimensions, proposed modifications and final items.

| **Item N** | **Initial item** | **Dimension** | **Proposed modifications: revised wording and lexical-syntactic reformulation** | **Final item** |
| --- | --- | --- | --- | --- |
| Q1 | I believe my heart disease is serious. | Beliefs | Exclusion of the verb form “I believe” **Reason for exclusion:** semantic redundancy, information not essential to the overall understanding of the item | My heart disease is serious. |
| Q2 | I believe that taking care of myself is important for managing my heart disease. | Beliefs | Exclusion of the verb form “I believe” **Reason for exclusion:** semantic redundancy, information not essential to the overall understanding of the item | Taking care of myself is important for managing my heart disease. |
| Q3 | I have trouble understanding why I take my medications. | Educational level | Exclusion of the statement “I have trouble” and change in the verb form of the transitive verb “understand.”  **Reason for exclusion**: to improve the clarity of the item. | I understand why I take my medications. |
| Q6 | I believe that skipping my medications every once in a while does not have serious effects on my health. | Risk perception | Exclusion of the verb form “I believe” **Reason for exclusion:** semantic redundancy, information not essential to the overall understanding of the item | Skipping my medication every once in a while doesn’t have serious consequences for my health. |
| Q8 | I believe that if I don’t take my medications correctly, I may suffer complications. | Risk perception | Exclusion of the verb form “I believe” **Reason for exclusion:** semantic redundancy, information not essential to the overall understanding of the item | If I don’t take my medication correctly, I may suffer from complications. |
| Q10 | If I stop taking my medications, it’s because I believe they may be harmful to me. | Fear | **Change in item response scale:**  From frequency to level of agreement | If I stop taking my medications, it’s because I believe they may be harmful to me. |
| Q11 | I believe that medications are a business for pharmaceutical companies. | Beliefs | Exclusion of the verb form “I believe”  **Reason for exclusion:** semantic redundancy, information not essential to the overall understanding of the item  **Change in the item’s location:** between the middle and the end of the questionnaire (item 11) to minimize the patient’s mistrust on beginning to complete the questionnaire | Medications are a business for pharmaceutical companies. |
| Q13 | Taking several pills a day makes it more difficult to take my medication properly. | Number of pills | Exclusion of the adverb “more”  **Reason for exclusion:** It implies a comparison with something that is not explicitly stated in the item. | Taking several pills a day makes it difficult to take my medication properly. |
| Q18 | I believe that the medications I take work well for me and are really helping me feel better. | Perception of treatment | Exclusion of the verb form “I believe”  **Reason for exclusion:** semantic redundancy, information not essential to the overall understanding of the item | The medications I take work well for me and are really helping me feel better. |
| Q20 | I use reminder systems (for example, pill boxes, notes, alarms) to remember to take my medication. | Reminder systems | Replacement of the verb form “I use” with the phrase “are useful”  **Reason for inclusion:** to improve the clarity of the item. | Reminder systems (for example pill boxes, notes, alarms) are useful for remembering to take my medication. |
| Q21 | I know the names of the medications I take. | Knowledge of medication | Inclusion of the indefinite pronoun “all”  **Reason for inclusion:** to improve the clarity of the item. | I know the names of all the medications I take. |
| Q24 | I have a good relationship with my cardiologist. | Relationship/ trust | Inclusion of the phrase “with the cardiology service as a whole”  **Reason for inclusion:** avoid social desirability bias in responses | I have a good relationship with the cardiology service as a whole. |
| Q27 | I am given enough time at my cardiologist's office. | Length of consultation | Inclusion of the noun phrase “the healthcare system” and change from “I am given enough time” to “My disease is given enough time”  **Reason for inclusion:** to avoid social desirability bias in responses | My disease is given enough time in the healthcare system. |
| Q31 | My heart disease is causing symptoms (for example, tachycardia, chest pain, shortness of breath, dizziness, general discomfort, fainting). | Lack of symptoms | Inclusion of the prepositional phrase “at present” and exclusion of the noun “symptoms”  **Reason for inclusion: /exclusion:** to avoid possible ambiguity in the interpretation of the item. | At present, my heart disease is causing, for example, tachycardia, chest pain, shortness of breath, dizziness, general discomfort, or fainting. |
| Q33 | Sometimes I just forget to take my medication. | Forgetfulness | Exclusion of the adverbs “sometimes,” and “just.”  **Reason for exclusion:** to improve the clarity of the item.  **Change in item response scale:** from level of agreement to frequency | I forget to take my medicine. |
| Q42 | My cardiologist explains to me the importance of taking my medication properly. | Explanation of adherence | Exclusion of the phrase “the importance of taking my medication properly” and inclusion of the phrase “how I should take my medication.”  **Reason for inclusion: /exclusion:** to avoid possible ambiguity in the interpretation of the item. | My cardiologist explains to me how I should take my medication. |

## Supplementary Table 2. Characteristics of patients undergoing cognitive interviews

| **Variable** | | **n** |  | **%** |
| --- | --- | --- | --- | --- |
| Sex | Male | 3 |  | 42.9 |
|  | Female | 4 |  | 57.1 |
| Age range | 40-49-years | 1 |  | 14.2 |
|  | 50-59-years | 3 |  | 42.9 |
|  | 60-69 years | 3 |  | 42.9 |
| Educational level | Higher education | 3 |  | 42.8 |
|  | Secondary education | 2 |  | 28.6 |
|  | Primary education | 2 |  | 28.6 |
|  | No schooling | 0 |  | 0.0 |
| Town/city | Alicante | 1 |  | 14.2 |
|  | San Juan de Alicante | 2 |  | 28.7 |
|  | El Campello | 3 |  | 42.9 |
|  | Valencia | 1 |  | 14.2 |

## Supplementary Table 3. Prevalence of comorbidities among participants (n=309)

| **Comorbidity** | **n** | **%** |
| --- | --- | --- |
| Hypertension | 205 | 66.3% |
| Diabetes | 96 | 31.1% |
| Dyslipidemia | 225 | 72.8% |
| Family history of cardiovascular disease | 2 | 0.6% |
| Atrial fibrillation | 10 | 3.2% |
| Heart failure | 14 | 4.5% |
| Valvular heart disease | 7 | 2.3% |
| Cardiomyopathy | 6 | 1.9% |
| Stroke | 15 | 4.9% |
| Aortic disease | 2 | 0.6% |
| Peripheral arterial disease | 17 | 5.5% |
| Cancer | 16 | 5.2% |
| Dementia | 1 | 0.3% |
| Chronic kidney disease | 17 | 5.5% |
| COPD and/or sleep apnea-hypopnea syndrome | 29 | 9.4% |
| Liver disease | 6 | 1.9% |

COPD: chronic obstructive pulmonary disease

## Supplementary Table 4.

## Ongoing pharmacological treatments in participants (n = 309)

|  | **Drug** | **n** | **%** |
| --- | --- | --- | --- |
| Antiplatelets | | 35 | 11.3% |
| Anticoagulants | | 15 | 4.9% |
| Beta-blockers | | 204 | 66.0% |
| Nitroglycerin therapy | | 15 | 4.9% |
| Calcium channel blockers | | 64 | 20.7% |
| ACE inhibitors | | 240 | 77.7% |
| Ivabradine | | 14 | 4.5% |
| Ranolazine | | 15 | 4.9% |
| Diuretics | | 88 | 28.5% |
| Insulin | | 25 | 8.1% |
| Metformin | | 74 | 23.9% |
| SGLT-2 inhibitors | | 83 | 26.9% |
| GLP-1 agonists | | 20 | 6.5% |
| Anti-diabetics | | 28 | 9.1% |
| Chemotherapy | | 1 | 0.3% |
| NSAIDs | | 1 | 0.3% |
| Hormone replacement therapy | | 1 | 0.3% |
| Cilostazol | | 4 | 1.3% |

ACE: angiotensin-converting-enzyme; GLP-1: glucagon-like peptide-1; NSAID: non-steroidal anti-inflammatory drugs; SGLT-2: sodium-glucose cotransporter 2

## Supplementary Table 5. Prevalence of non-adherence to statins, as estimated using the gold standard, according to sociodemographic and clinical variables

|  | | **Partially adherent/ adherent** | | | **Non-adherent** | |  |
| --- | --- | --- | --- | --- | --- | --- | --- |
| **Variables** | | | **n** | **%** | **n** | **%** | **p- value*** |
| Sex | Female | | 86 | 85.1% | 15 | 14.9% | 0.87 |
|  | Male | | 174 | 83.7% | 34 | 16.3% |  |
| Nationality | Spanish | | 247 | 84.3% | 46 | 15.7% | 0.73 |
|  | Other | | 13 | 81.3% | 3 | 18.8% |  |
| Educational attainment | ≤ Primary school | | 109 | 77.3% | 32 | 22.7% | 0.003 |
|  | Secondary school | | 78 | 94.0% | 5 | 6.0% |  |
|  | Higher education | | 73 | 85.9% | 12 | 14.1% |  |
| Employment status | Employed | | 71 | 85.5% | 12 | 14.5% | 0.78 |
|  | Unemployed | | 21 | 77.8% | 6 | 22.2% |  |
|  | Retired | | 157 | 84.4% | 29 | 15.6% |  |
|  | Homemaker | | 11 | 84.6% | 2 | 15.4% |  |
| Marital status | Single | | 21 | 91.3% | 2 | 8.7% | 0.29 |
|  | Married/ attached | | 194 | 85.1% | 34 | 14.9% |  |
|  | Separated/divorced | | 32 | 74.4% | 11 | 25.6% |  |
|  | Widowed | | 13 | 86.7% | 2 | 13.3% |  |
| Net monthly household income | < 800 € | | 40 | 80.0% | 10 | 20.0% | 0.47 |
|  | 800-1499 € | | 87 | 83.7% | 17 | 16.3% |  |
|  | 1500-3000 € | | 80 | 83.3% | 16 | 16.7% |  |
|  | > 3000 € | | 50 | 90.9% | 5 | 9.1% |  |
|  | NA | | 3 | 75.0% | 1 | 25.0% |  |
| Living situation | Lives alone | | 41 | 82.0% | 9 | 18.0% | 0.67 |
|  | Lives with someone | | 219 | 84.6% | 40 | 15.4% |  |
| Caregiver | No | | 238 | 84.4% | 44 | 15.6% | 0.78 |
|  | Yes | | 22 | 81.5% | 5 | 18.5% |  |
| Self-perceived health status | Very poor | | 3 | 75.0% | 1 | 25.0% | 0.65 |
|  | Poor | | 13 | 76.5% | 4 | 23.5% |  |
|  | Fair | | 83 | 86.5% | 13 | 13.5% |  |
|  | Good | | 129 | 84.3% | 24 | 15.7% |  |
|  | Very good | | 32 | 82.1% | 7 | 17.9% |  |
| Tobacco use | Current smoker | | 27 | 77.1% | 8 | 22.9% | 0.47 |
|  | Ex-smoker | | 150 | 84.7% | 27 | 15.3% |  |
|  | Never smoker | | 83 | 85.6% | 14 | 14.4% |  |
| Alcohol use | Abstainer | | 107 | 84.9% | 19 | 15.1% | 0.50 |
|  | Once weekly | | 98 | 81.7% | 22 | 18.3% |  |
|  | Several times weekly | | 35 | 83.3% | 7 | 16.7% |  |
|  | Every day | | 20 | 95.2% | 1 | 4.8% |  |
| Leisure time physical activity | Sedentary | | 39 | 84.8% | 7 | 15.2% | 0.31 |
|  | Occasional physical activity | | 94 | 81.7% | 21 | 18.3% |  |
|  | Several times a month | | 93 | 83.0% | 19 | 17.0% |  |
|  | Several times a week | | 34 | 94.4% | 2 | 5.6% |  |
| Main activity during the day | Sitting | | 83 | 80.6% | 20 | 19.4% | 0.14 |
|  | Standing | | 107 | 85.6% | 18 | 14.4% |  |
|  | Walking with weight | | 65 | 89.0% | 8 | 11.0% |  |
|  | Strenuous tasks | | 5 | 62.5% | 3 | 37.5% |  |
| Hypertension | No | | 85 | 81.7% | 19 | 18.3% | 0.41 |
|  | Yes | | 175 | 85.4% | 30 | 14.6% |  |
| Diabetes | No | | 175 | 82.2% | 38 | 17.8% | 0.18 |
|  | Yes | | 85 | 88.5% | 11 | 11.5% |  |
| Dyslipidemia | No | | 70 | 83.3% | 14 | 16.7% | 0.86 |
|  | Yes | | 190 | 84.4% | 35 | 15.6% |  |
| Atrial fibrillation | No | | 252 | 84.3% | 47 | 15.7% | 0.66 |
|  | Yes | | 8 | 80.0% | 2 | 20.0% |  |
| Heart failure | No | | 249 | 84.4% | 46 | 15.6% | 0.47 |
|  | Yes | | 11 | 78.6% | 3 | 21.4% |  |
| Stroke | No | | 246 | 83.7% | 48 | 16.3% | 0.48 |
|  | Yes | | 14 | 93.3% | 1 | 6.7% |  |
| Peripheral arterial disease | No | | 246 | 84.2% | 46 | 15.8% | 0.74 |
|  | Yes | | 14 | 82.4% | 3 | 17.6% |  |
| Chronic kidney disease | No | | 244 | 83.6% | 48 | 16.4% | 0.49 |
|  | Yes | | 16 | 94.1% | 1 | 5.9% |  |
| COPD and/or sleep apnea-hypopnea syndrome | No | | 236 | 84.3% | 44 | 15.7% | 0.79 |
|  | Yes | | 24 | 82.8% | 5 | 17.2% |  |

* Fisher’s exact test

## Supplementary Table 6. Expert consensus dichotomization of item responses: assignment of Likert scale (5-point) responses to the category "suggestive of non-adherence".

| **Item** | **Strongly agree** | **Agree** | **Neither agree nor disagree** | **Disagree** | **Strongly disagree** |
| --- | --- | --- | --- | --- | --- |
| Q1 - My heart disease is serious. |  |  |  |  |  |
| Q2 - Taking care of myself is important for managing my heart disease. |  |  |  |  |  |
| Q3 - I understand why I take my medications. |  |  |  |  |  |
| Q4 – I feel sad or depressed. |  |  |  |  |  |
| Q5 - My disease frightens me. |  |  |  |  |  |
| Q6 - Skipping my medication every once in a while doesn’t have serious consequences for my health. |  |  |  |  |  |
| Q7 – When I’m feeling better, I stop taking my medication or I take less of it. |  |  |  |  |  |
| Q8 - If I don’t take my medication correctly, I may suffer from complications. |  |  |  |  |  |
| Q9 – Nobody helps me take my medication. |  |  |  |  |  |
| Q10 - If I stop taking my medications, it’s because I believe they may be harmful to me. |  |  |  |  |  |
| Q11 - Medications are a business for pharmaceutical companies. |  |  |  |  |  |
| Q12 - I know what the medications I take are for. |  |  |  |  |  |
| Q13 - Taking several pills a day makes it difficult to take my medication properly. |  |  |  |  |  |
| Q14 - Buying the medications I need for my heart condition is a big financial strain on me. |  |  |  |  |  |
| Q15 – Sometimes I run out of medication. |  |  |  |  |  |
| Q16 - The change in the format (color, shape, and/or brand name) of my medication confuses me when it comes to taking my medication. |  |  |  |  |  |
| Q17 - Taking medication just once a day would make it easier for me to take it correctly. |  |  |  |  |  |
| Q18 - The medications I take work well for me and are really helping me feel better and stay healthy. |  |  |  |  |  |
| Q19 - Taking my medication prevents my heart disease from getting worse. |  |  |  |  |  |
| Q20 - Reminder systems (for example pill boxes, notes, alarms) are useful for remembering to take my medication. |  |  |  |  |  |
| Q21 - I know the names of all the medications I take. |  |  |  |  |  |
| Q22 - Taking my medication causes some unpleasant side effects. |  |  |  |  |  |
| Q23 - Health professionals keep my preferences and opinions in mind to treat my disease. |  |  |  |  |  |
| Q24 - I have a good relationship with the cardiology service as a whole. |  |  |  |  |  |
| Q25 - I know what cholesterol levels I should have to keep my heart disease under control. |  |  |  |  |  |
| Q26 - I know what blood pressure levels I should have to keep my heart disease under control. |  |  |  |  |  |
| Q27 - My disease is given enough time in the healthcare system. |  |  |  |  |  |
| Q28 - Another health professional (nurse, pharmacist, family doctor) helps me to follow my treatment correctly. |  |  |  |  |  |
| Q29 - Since I’ve had heart problems, I visit my cardiologist at least once a year. |  |  |  |  |  |
| Q30 - My heart condition is not dangerous to my health. |  |  |  |  |  |
| Q31 - At present, my heart disease is causing, for example, tachycardia, chest pain, shortness of breath, dizziness, general discomfort, or fainting. |  |  |  |  |  |

## Supplementary Table 6, *continued*. Dichotomization of item responses according to frequency*.

| **Item** | **Always** | **Almost always** | **Sometimes** | **Seldom** | **Never** |
| --- | --- | --- | --- | --- | --- |
| Q32 - I leave medication somewhere other than my home (e.g., work, car, purse, wallet) in case I forget to take it at home. |  |  |  |  |  |
| Q33 - I forget to take my medicine. |  |  |  |  |  |
| Q34 - When I leave my usual environment (for example, if I am away from home, on vacations, out of town), I forget to take my medication. |  |  |  |  |  |
| Q35 - I prefer natural remedies to conventional medicines. |  |  |  |  |  |
| Q36 - Picking up my medication from the pharmacy is difficult for me. |  |  |  |  |  |
| Q37 - Visiting my cardiologist is difficult for me, for example, because it’s difficult to get an appointment, they don’t answer the phone, it is far from my home, etc. |  |  |  |  |  |
| Q38 - I take medication more than once a day. |  |  |  |  |  |
| Q39 - I have trouble swallowing my pills. |  |  |  |  |  |
| Q40 - In the last month, I have felt bad or have had an unexpected symptom due to the medications I take. |  |  |  |  |  |
| Q41 - I notice that the medication I take negatively affects my sex life. |  |  |  |  |  |
| Q42 - My cardiologist explains to me how I should take my medication. |  |  |  |  |  |

*Each questionnaire item was evaluated on a five-point Likert scale ranging from strongly agree to strongly disagree. The orange-shaded cells indicate the response categories considered suggestive of non-adherence. The dichotomization scheme was not identical across items, as the interpretation of agreement versus disagreement varied according to the item content.

## Supplementary table 7. Frequency of dichotomized responses for each study item.

|  | Missings n(%)* |  |  | n | % |
| --- | --- | --- | --- | --- | --- |
| Q1 | 1 (0.3%) | strongly agree, agree | | 235 | 76.1% |
|  |  | neither agree nor disagree, disagree, strongly disagree | | 74 | 23.9% |
| Q2 | 0 (0.0%) | strongly agree, agree | | 303 | 98.1% |
|  |  | neither agree nor disagree, disagree, strongly disagree | | 6 | 1.9% |
| Q3 | 0 (0.0%) | strongly agree, agree | | 304 | 98.4% |
|  |  | neither agree nor disagree, disagree, strongly disagree | | 5 | 1.6% |
| Q4 | 2 (0.6%) | strongly agree, agree, neither agree nor disagree | | 139 | 45.0% |
|  |  | disagree, strongly disagree | | 170 | 55.0% |
| Q5 | 0 (0.0%) | strongly agree, agree, neither agree nor disagree | | 187 | 60.5% |
|  |  | disagree, strongly disagree | | 122 | 39.5% |
| Q6 | 2 (0.6%) | strongly agree, agree, neither agree nor disagree | | 145 | 46.9% |
|  |  | disagree, strongly disagree | | 164 | 53.1% |
| Q7 | 0 (0.0%) | strongly agree, agree, neither agree nor disagree | | 27 | 8.7% |
|  |  | disagree, strongly disagree | | 282 | 91.3% |
| Q8 | 0 (0.0%) | strongly agree, agree | | 271 | 87.7% |
|  |  | neither agree nor disagree, disagree, strongly disagree | | 38 | 12.3% |
| Q9 | 1 (0.3%) | strongly agree, agree, neither agree nor disagree | | 236 | 76.4% |
|  |  | disagree, strongly disagree | | 73 | 23.6% |
| Q10 | 1 (0.3%) | strongly agree, agree, neither agree nor disagree | | 116 | 37.5% |
|  |  | disagree, strongly disagree | | 193 | 62.5% |
| Q11 | 0 (0.0%) | strongly agree, agree, neither agree nor disagree | | 169 | 54.7% |
|  |  | disagree, strongly disagree | | 140 | 45.3% |
| Q12 | 0 (0.0%) | strongly agree, agree | | 281 | 90.9% |
|  |  | neither agree nor disagree, disagree, strongly disagree | | 28 | 9.1% |
| Q13 | 0 (0.0%) | strongly agree, agree, neither agree nor disagree | | 96 | 31.1% |
|  |  | disagree, strongly disagree | | 213 | 68.9% |
| Q14 | 0 (0.0%) | strongly agree, agree, neither agree nor disagree | | 121 | 39.2% |
|  |  | disagree, strongly disagree | | 188 | 60.8% |
| Q15 | 0 (0.0%) | strongly agree, agree, neither agree nor disagree | | 43 | 13.9% |
|  |  | disagree, strongly disagree | | 266 | 86.1% |
| Q16 | 0 (0.0%) | strongly agree, agree, neither agree nor disagree | | 102 | 33.0% |
|  |  | disagree, strongly disagree | | 207 | 67.0% |
| Q17 | 0 (0.0%) | strongly agree, agree, neither agree nor disagree | | 200 | 64.7% |
|  |  | disagree, strongly disagree | | 109 | 35.3% |
| Q18 | 0 (0.0%) | strongly agree, agree | | 273 | 88.3% |
|  |  | neither agree nor disagree, disagree, strongly disagree | | 36 | 11.7% |
| Q19 | 0 (0.0%) | strongly agree, agree | | 277 | 89.6% |
|  |  | neither agree nor disagree, disagree, strongly disagree | | 32 | 10.4% |
| Q20 | 0 (0.0%) | strongly agree, agree | | 210 | 68.0% |
|  |  | neither agree nor disagree, disagree, strongly disagree | | 99 | 32.0% |
| Q21 | 0 (0.0%) | strongly agree, agree | | 231 | 74.8% |
|  |  | neither agree nor disagree, disagree, strongly disagree | | 78 | 25.2% |
| Q22 | 0 (0.0%) | strongly agree, agree, neither agree nor disagree | | 110 | 35.6% |
|  |  | disagree, strongly disagree | | 199 | 64.4% |
| Q23 | 0 (0.0%) | strongly agree, agree | | 223 | 72.2% |
|  |  | neither agree nor disagree, disagree, strongly disagree | | 86 | 27.8% |
| Q24 | 0 (0.0%) | strongly agree, agree | | 289 | 93.5% |
|  |  | neither agree nor disagree, disagree, strongly disagree | | 20 | 6.5% |
| Q25 | 0 (0.0%) | strongly agree, agree | | 230 | 74.4% |
|  |  | neither agree nor disagree, disagree, strongly disagree | | 79 | 25.6% |
| Q26 | 0 (0.0%) | strongly agree, agree | | 270 | 87.4% |
|  |  | neither agree nor disagree, disagree, strongly disagree | | 39 | 12.6% |
| Q27 | 0 (0.0%) | strongly agree, agree | | 245 | 79.3% |
|  |  | neither agree nor disagree, disagree, strongly disagree | | 64 | 20.7% |
| Q28 | 1 (0.3%) | strongly agree, agree | | 175 | 56.6% |
|  |  | neither agree nor disagree, disagree, strongly disagree | | 134 | 43.4% |
| Q29 | 1 (0.3%) | strongly agree, agree | | 293 | 94.8% |
|  |  | neither agree nor disagree, disagree, strongly disagree | | 16 | 5.2% |
| Q30 | 1 (0.3%) | strongly agree, agree, neither agree nor disagree | | 114 | 36.9% |
|  |  | disagree, strongly disagree | | 195 | 63.1% |
| Q31 | 1 (0.3%) | strongly agree, agree, neither agree nor disagree | | 107 | 34.6% |
|  |  | disagree, strongly disagree | | 202 | 65.4% |
| Q32 | 1 (0.3%) | always, almost always, sometimes | | 72 | 23.3% |
|  |  | seldom, never | | 237 | 76.7% |
| Q33 | 1 (0.3%) | always, almost always, sometimes | | 13 | 4.2% |
|  |  | seldom, never | | 296 | 95.8% |
| Q34 | 1 (0.3%) | always, almost always, sometimes | | 17 | 5.5% |
|  |  | seldom, never | | 292 | 94.5% |
| Q35 | 1 (0.3%) | always, almost always, sometimes, seldom | | 75 | 24.3% |
|  |  | never | | 234 | 75.7% |
| Q36 | 2 (0.6%) | always, almost always, sometimes | | 27 | 8.7% |
|  |  | seldom, never | | 282 | 91.3% |
| Q37 | 2 (0.6%) | always, almost always, sometimes | | 55 | 17.8% |
|  |  | seldom, never | | 254 | 82.2% |
| Q38 | 2 (0.6%) | always, almost always, sometimes | | 266 | 86.1% |
|  |  | seldom, never | | 43 | 13.9% |
| Q39 | 2 (0.6%) | always, almost always, sometimes | | 19 | 6.1% |
|  |  | seldom, never | | 290 | 93.9% |
| Q40 | 2 (0.6%) | always, almost always, sometimes | | 34 | 11.0% |
|  |  | seldom, never | | 275 | 89.0% |
| Q41 | 4 (1.2%) | always, almost always, sometimes | | 87 | 28.2% |
|  |  | seldom, never | | 222 | 71.8% |
| Q42 | 3 (0.9%) | always, almost always, sometimes | | 293 | 94.8% |
|  |  | seldom, never | | 16 | 5.2% |

* Number of missings before imputation

## Supplementary table 8. Association between dichotomized study items and adherence to statin treatment measured by the pharmacokinetic reference method.

| Item |  | *Adherent* | | *Non-adherent* | | p-value* |
| --- | --- | --- | --- | --- | --- | --- |
|  |  | *n* | *%* | *n* | *%* |  |
| Q1 | strongly agree, agree | 200 | 85,1% | 35 | 14,9% | 0.41 |
|  | neither agree nor disagree, disagree, strongly disagree | 60 | 81,1% | 14 | 18,9% |  |
| Q2 | strongly agree, agree | 256 | 84,5% | 47 | 15,5% | 0.24 |
|  | neither agree nor disagree, disagree, strongly disagree | 4 | 66,7% | 2 | 33,3% |  |
| Q3 | strongly agree, agree | 256 | 84,2% | 48 | 15,8% | 0.80 |
|  | neither agree nor disagree, disagree, strongly disagree | 4 | 80,0% | 1 | 20,0% |  |
| Q4 | strongly agree, agree, neither agree nor disagree | 117 | 84,2% | 22 | 15,8% | 0.99 |
|  | disagree, strongly disagree | 143 | 84,1% | 27 | 15,9% |  |
| Q5 | strongly agree, agree, neither agree nor disagree | 153 | 81,8% | 34 | 18,2% | 0.17 |
|  | disagree, strongly disagree | 107 | 87,7% | 15 | 12,3% |  |
| Q6 | strongly agree, agree, neither agree nor disagree | 116 | 80,0% | 29 | 20,0% | 0.033 |
|  | disagree, strongly disagree | 144 | 87,8% | 20 | 12,2% |  |
| Q7 | strongly agree, agree, neither agree nor disagree | 23 | 85,2% | 4 | 14,8% | 0.88 |
|  | disagree, strongly disagree | 237 | 84,0% | 45 | 16,0% |  |
| Q8 | strongly agree, agree | 231 | 85,2% | 40 | 14,8% | 0.16 |
|  | neither agree nor disagree, disagree, strongly disagree | 29 | 76,3% | 9 | 23,7% |  |
| Q9 | strongly agree, agree, neither agree nor disagree | 197 | 83,5% | 39 | 16,5% | 0.56 |
|  | disagree, strongly disagree | 63 | 86,3% | 10 | 13,7% |  |
| Q10 | strongly agree, agree, neither agree nor disagree | 98 | 84,5% | 18 | 15,5% | 0.90 |
|  | disagree, strongly disagree | 162 | 83,9% | 31 | 16,1% |  |
| Q11 | strongly agree, agree, neither agree nor disagree | 142 | 84,0% | 27 | 16,0% | 0.95 |
|  | disagree, strongly disagree | 118 | 84,3% | 22 | 15,7% |  |
| Q12 | strongly agree, agree | 234 | 83,3% | 47 | 16,7% | 0.19 |
|  | neither agree nor disagree, disagree, strongly disagree | 26 | 92,9% | 2 | 7,1% |  |
| Q13 | strongly agree, agree, neither agree nor disagree | 81 | 84,4% | 15 | 15,6% | 0.94 |
|  | disagree, strongly disagree | 179 | 84,0% | 34 | 16,0% |  |
| Q14 | strongly agree, agree, neither agree nor disagree | 99 | 81,8% | 22 | 18,2% | 0.37 |
|  | disagree, strongly disagree | 161 | 85,6% | 27 | 14,4% |  |
| Q15 | strongly agree, agree, neither agree nor disagree | 36 | 83,7% | 7 | 16,3% | 0.94 |
|  | disagree, strongly disagree | 224 | 84,2% | 42 | 15,8% |  |
| Q16 | strongly agree, agree, neither agree nor disagree | 87 | 85,3% | 15 | 14,7% | 0.70 |
|  | disagree, strongly disagree | 173 | 83,6% | 34 | 16,4% |  |
| Q17 | strongly agree, agree, neither agree nor disagree | 173 | 86,5% | 27 | 13,5% | 0.12 |
|  | disagree, strongly disagree | 87 | 79,8% | 22 | 20,2% |  |
| Q18 | strongly agree, agree | 230 | 84,2% | 43 | 15,8% | 0.89 |
|  | neither agree nor disagree, disagree, strongly disagree | 30 | 83,3% | 6 | 16,7% |  |
| Q19 | strongly agree, agree | 233 | 84,1% | 44 | 15,9% | 0.97 |
|  | neither agree nor disagree, disagree, strongly disagree | 27 | 84,4% | 5 | 15,6% |  |
| Q20 | strongly agree, agree | 183 | 87,1% | 27 | 12,9% | 0.035 |
|  | neither agree nor disagree, disagree, strongly disagree | 77 | 77,8% | 22 | 22,2% |  |
| Q21 | strongly agree, agree | 193 | 83,5% | 38 | 16,5% | 0.62 |
|  | neither agree nor disagree, disagree, strongly disagree | 67 | 85,9% | 11 | 14,1% |  |
| Q22 | strongly agree, agree, neither agree nor disagree | 91 | 82,7% | 19 | 17,3% | 0.61 |
|  | disagree, strongly disagree | 169 | 84,9% | 30 | 15,1% |  |
| Q23 | strongly agree, agree | 185 | 83,0% | 38 | 17,0% | 0.36 |
|  | neither agree nor disagree, disagree, strongly disagree | 75 | 87,2% | 11 | 12,8% |  |
| Q24 | strongly agree, agree | 243 | 84,1% | 46 | 15,9% | 0.91 |
|  | neither agree nor disagree, disagree, strongly disagree | 17 | 85,0% | 3 | 15,0% |  |
| Q25 | strongly agree, agree | 193 | 83,9% | 37 | 16,1% | 0.85 |
|  | neither agree nor disagree, disagree, strongly disagree | 67 | 84,8% | 12 | 15,2% |  |
| Q26 | strongly agree, agree | 230 | 85,2% | 40 | 14,8% | 0.19 |
|  | neither agree nor disagree, disagree, strongly disagree | 30 | 76,9% | 9 | 23,1% |  |
| Q27 | strongly agree, agree | 209 | 85,3% | 36 | 14,7% | 0.27 |
|  | neither agree nor disagree, disagree, strongly disagree | 51 | 79,7% | 13 | 20,3% |  |
| Q28 | strongly agree, agree | 151 | 86,3% | 24 | 13,7% | 0.24 |
|  | neither agree nor disagree, disagree, strongly disagree | 109 | 81,3% | 25 | 18,7% |  |
| Q29 | strongly agree, agree | 247 | 84,3% | 46 | 15,7% | 0.75 |
|  | neither agree nor disagree, disagree, strongly disagree | 13 | 81,3% | 3 | 18,8% |  |
| Q30 | strongly agree, agree, neither agree nor disagree | 95 | 83,3% | 19 | 16,7% | 0.77 |
|  | disagree, strongly disagree | 165 | 84,6% | 30 | 15,4% |  |
| Q31 | strongly agree, agree, neither agree nor disagree | 88 | 82,2% | 19 | 17,8% | 0.51 |
|  | disagree, strongly disagree | 172 | 85,1% | 30 | 14,9% |  |
| Q32 | always, almost always, sometimes | 58 | 80,6% | 14 | 19,4% | 0.34 |
|  | seldom, never | 202 | 85,2% | 35 | 14,8% |  |
| Q33 | always, almost always, sometimes | 8 | 61,5% | 5 | 38,5% | 0.023 |
|  | seldom, never | 252 | 85,1% | 44 | 14,9% |  |
| Q34 | always, almost always, sometimes | 15 | 88,2% | 2 | 11,8% | 0.64 |
|  | seldom, never | 245 | 83,9% | 47 | 16,1% |  |
| Q35 | always, almost always, sometimes, seldom | 63 | 84,0% | 12 | 16,0% | 0.97 |
|  | never | 197 | 84,2% | 37 | 15,8% |  |
| Q36 | always, almost always, sometimes | 20 | 74,1% | 7 | 25,9% | 0.13 |
|  | seldom, never | 240 | 85,1% | 42 | 14,9% |  |
| Q37 | always, almost always, sometimes | 45 | 81,8% | 10 | 18,2% | 0.60 |
|  | seldom, never | 215 | 84,6% | 39 | 15,4% |  |
| Q38 | always, almost always, sometimes | 225 | 84,6% | 41 | 15,4% | 0.60 |
|  | seldom, never | 35 | 81,4% | 8 | 18,6% |  |
| Q39 | always, almost always, sometimes | 16 | 84,2% | 3 | 15,8% | 0.99 |
|  | seldom, never | 244 | 84,1% | 46 | 15,9% |  |
| Q40 | always, almost always, sometimes | 26 | 76,5% | 8 | 23,5% | 0.19 |
|  | seldom, never | 234 | 85,1% | 41 | 14,9% |  |
| Q41 | always, almost always, sometimes | 73 | 83,9% | 14 | 16,1% | 0.94 |
|  | seldom, never | 187 | 84,2% | 35 | 15,8% |  |
| Q42 | always, almost always, sometimes | 247 | 84,3% | 46 | 15,7% | 0.75 |
|  | seldom, never | 13 | 81,3% | 3 | 18,8% |  |

* Chi-squared test

## Supplementary table 9. Measures of diagnostic accuracy for predictive model in men

| Cutoff | Overall detection accuracy | Youden index | Sensitivity | (95% CI) | Specificity | (95% CI) | PPV | (95% CI) | NPV | (95% CI) | LR+ | (95% CI) | LR− | (95% CI) |
| --- | --- | --- | --- | --- | --- | --- | --- | --- | --- | --- | --- | --- | --- | --- |
| 0.10 | 0.56 | 0.33 | 0.82 | (0.69-0.95) | 0.51 | (0.44-0.58) | 0.25 | (0.17-0.33) | 0.94 | (0.89-0.99) | 1.67 | (1.34-2.08) | 0.35 | (0.17-0.73) |
| 0.15 | 0.56 | 0.33 | 0.82 | (0.69-0.95) | 0.51 | (0.44-0.58) | 0.25 | (0.17-0.33) | 0.94 | (0.89-0.99) | 1.67 | (1.34-2.08) | 0.35 | (0.17-0.73) |
| 0.20 | 0.79 | 0.30 | 0.44 | (0.27-0.61) | 0.86 | (0.81-0.91) | 0.38 | (0.23-0.53) | 0.89 | (0.84-0.94) | 3.14 | (1.86-5.31) | 0.65 | (0.48-0.88) |
| 0.25 | 0.78 | 0.27 | 0.41 | (0.24-0.58) | 0.86 | (0.81-0.91) | 0.36 | (0.21-0.51) | 0.88 | (0.83-0.93) | 2.93 | (1.70-5.04) | 0.69 | (0.52-0.92) |
| 0.30 | 0.78 | 0.27 | 0.41 | (0.24-0.58) | 0.86 | (0.81-0.91) | 0.36 | (0.21-0.51) | 0.88 | (0.83-0.93) | 2.93 | (1.70-5.04) | 0.69 | (0.52-0.92) |
| 0.35 | 0.83 | 0.07 | 0.09 | (0.00-0.19) | 0.98 | (0.96-1.00) | 0.43 | (0.06-0.80) | 0.85 | (0.8-0.90) | 4.50 | (1.05-19.21) | 0.93 | (0.84-1.04) |
| 0.40 | 0.84 | 0.07 | 0.09 | (0.00-0.19) | 0.98 | (0.96-1.00) | 0.50 | (0.10-0.90) | 0.85 | (0.8-0.90) | 4.50 | (0.95-21.32) | 0.93 | (0.84-1.04) |
| 0.45 | 0.84 | 0.05 | 0.06 | (0.00-0.14) | 0.99 | (0.98-1.00) | 0.50 | (0.01-0.99) | 0.84 | (0.79-0.89) | 6.00 | (0.87-41.15) | 0.95 | (0.87-1.04) |
| 0.50 | 0.84 | 0.05 | 0.06 | (0.00-0.14) | 0.99 | (0.98-1.00) | 0.50 | (0.01-0.99) | 0.84 | (0.79-0.89) | 6.00 | (0.87-41.15) | 0.95 | (0.87-1.04) |
| 0.55 | 0.84 | 0.05 | 0.06 | (0.00-0.14) | 0.99 | (0.98-1.00) | 0.50 | (0.01-0.99) | 0.84 | (0.79-0.89) | 6.00 | (0.87-41.15) | 0.95 | (0.87-1.04) |

CI: confidence interval; LR+: positive likelihood ratio; LR−: negative likelihood ratios; NPV: negative predictive value; PPV: positive predictive value.

## Supplementary table 10. Measures of diagnostic accuracy for predictive model in women

| Cutoff | Overall detection accuracy | Youden index | Sensitivity | (95% CI) | Specificity | (95% CI) | PPV | (95% CI) | NPV | (95% CI) | LR+ | (95% CI) | LR− | (95% CI) |
| --- | --- | --- | --- | --- | --- | --- | --- | --- | --- | --- | --- | --- | --- | --- |
| 0.10 | 0.46 | 0.25 | 0.87 | (0.70-1.04) | 0.38 | (0.28-0.48) | 0.20 | (0.10-0.30) | 0.94 | (0.86-1.02) | 1.40 | (1.08-1.81) | 0.34 | (0.09-1.27) |
| 0.15 | 0.75 | 0.38 | 0.60 | (0.35-0.85) | 0.78 | (0.69-0.87) | 0.32 | (0.15-0.49) | 0.92 | (0.86-0.98) | 2.73 | (1.54-4.84) | 0.51 | (0.27-0.96) |
| 0.20 | 0.75 | 0.38 | 0.60 | (0.35-0.85) | 0.78 | (0.69-0.87) | 0.32 | (0.15-0.49) | 0.92 | (0.86-0.98) | 2.73 | (1.54-4.84) | 0.51 | (0.27-0.96) |
| 0.25 | 0.79 | 0.37 | 0.53 | (0.28-0.78) | 0.84 | (0.76-0.92) | 0.36 | (0.16-0.56) | 0.91 | (0.85-0.97) | 3.31 | (1.68-6.50) | 0.56 | (0.32-0.97) |
| 0.30 | 0.88 | 0.26 | 0.27 | (0.05-0.49) | 0.99 | (0.97-1.01) | 0.80 | (0.45-1.15) | 0.89 | (0.83-0.95) | 27.00 | (3.23-225.46) | 0.74 | (0.54-1.01) |
| 0.35 | 0.88 | 0.26 | 0.27 | (0.05-0.49) | 0.99 | (0.97-1.01) | 0.80 | (0.45-1.15) | 0.89 | (0.83-0.95) | 27.00 | (3.23-225.46) | 0.74 | (0.54-1.01) |
| 0.40 | 0.88 | 0.26 | 0.27 | (0.05-0.49) | 0.99 | (0.97-1.01) | 0.80 | (0.45-1.15) | 0.89 | (0.83-0.95) | 27.00 | (3.23-225.46) | 0.74 | (0.54-1.01) |
| 0.45 | 0.88 | 0.26 | 0.27 | (0.05-0.49) | 0.99 | (0.97-1.01) | 0.80 | (0.45-1.15) | 0.89 | (0.83-0.95) | 27.00 | (3.23-225.46) | 0.74 | (0.54-1.01) |

CI: confidence interval; LR+: positive likelihood ratio; LR−: negative likelihood ratios; NPV: negative predictive value; PPV: positive predictive value.

Supplemental Figures

Supplemental Figure S1. Calibration plot for predictive model.

Supplemental Figure S2. Decision curve for predictive model.

# References

1. Argimón Pallas JM, Jiménez Villa J. Métodos de investigación clínica y epidemiológica. 5th ed. Barcelona: Elsevier. (2019).
2. Jones TL, Baxter MA, Khanduja V. A quick guide to survey research. Ann R Coll Surg Engl. (2013) 95(1):5–7. doi: 10.1308/003588413X13511609956372
3. Casas Anguita J, Repullo Labrador JR, Donado Campos J. La encuesta como técnica de investigación. Elaboración de cuestionarios y tratamiento estadístico de los datos (II). Aten Primaria. (2003) 31(9):592–600. doi: 10.1016/s0212-6567(03)79222-1
4. Rosellini AJ, Brown TA. Developing and validating clinical questionnaires. Annu Rev Clin Psychol. (2021) 17:55–81. doi: 10.1146/annurev-clinpsy-081219-115343
5. León OG, Montero I. Métodos de investigación en psicología y educación: las tradiciones cuantitativa y cualitativa. 4th ed. Madrid: McGraw-Hill. (2020).
6. Rosellini AJ, Brown TA. Developing and validating clinical questionnaires. Annu Rev Clin Psychol. (2021) 17:55–81. doi: 10.1146/annurev-clinpsy-081219-115343
7. León OG, Montero I. Métodos de investigación en psicología y educación: las tradiciones cuantitativa y cualitativa. 4th ed. Madrid: McGraw-Hill. (2020).
8. van Teijlingen E, Hundley V. The importance of pilot studies. Nurs Stand. (2002) 16(40):33–36. doi: 10.7748/ns2002.06.16.40.33.c3214
9. Carbonell-Soliva Á, Nouni-García R, López-Pineda A, Cordero-Fort A, Pérez-Jover V, Quesada JA, et al. Opinions and perceptions of patients with cardiovascular disease on adherence: a qualitative study of focus groups. BMC Prim Care. (2024) 25(1):59. doi: 10.1186/s12875-024-02286-8
10. Kristiansen O, Vethe NT, Fagerland MW, Bergan S, Munkhaugen J, Husebye E, et al. A novel direct method to determine adherence to atorvastatin therapy in patients with coronary heart disease. Br J Clin Pharmacol. (2019) 85(12):2878–2885. doi: 10.1111/bcp.14122
11. Kanukula R, Salam A, Rodgers A, Kamel B. Pharmacokinetics of Rosuvastatin: A Systematic Review of Randomised Controlled Trials in Healthy Adults. Clin Pharmacokinet. 2021 Feb;60(2):165-175. doi: 10.1007/s40262-020-00978-9.
12. Ficha técnica Rosuvastatina Disponible en: <https://cima.aemps.es/cima/dochtml/ft/79819/FT_79819.html>. Último acceso: 25 November 2025
